# TRuE-XAI: causal and explainable ai framework for trustworthy corporate earnings growth forecasting

**DOI:** 10.3389/frai.2026.1885910

**Published:** 2026-07-27

**Authors:** Gopal Singh Jamnal

**Affiliations:** School of Built Environment, Engineering & Computing, Leeds Beckett University, Leeds, United Kingdom

**Keywords:** anchor explanations, causal inference, class-imbalanced learning, corporate finance, earnings growth forecasting, ensemble learning, Explainable Artificial Intelligence (XAI), hyperparameter optimization

## Abstract

Forecasting corporate earnings growth is fundamental to investment, credit, and regulatory decision-making. Existing forecasting approaches either rely on restrictive linear assumptions or provide limited interpretability, making them less suitable for high-stakes financial applications. This study proposes a transparent and causally informed framework for predicting future corporate earnings growth from financial statement data. We present TRuE-XAI (Transparent, Rule-based, and Explainable Artificial Intelligence), an integrated framework combining imbalance-aware ensemble learning, automated hyperparameter optimization, rule-based explainability, visual analytics, and causal inference. Random Forest, XGBoost, and LightGBM classifiers were optimized using Optuna and Hyperopt and evaluated with multiple class-balancing strategies, including SMOTE, ADASYN, TomekLinks, and Repeated Edited Nearest Neighbours (RENN). Experiments were conducted on real-world SEC-derived quarterly financial statement data from U.S. publicly listed firms covering 2014–2024. Model transparency was achieved through Anchor explanations, multi-metric feature-importance analysis, SilVA visual analytics, and causal effect estimation using DoWhy and EconML. The best-performing configuration, TomekLinks–XGBoost, achieved an F1-score of 0.467 and accuracy of 0.849 on the real SEC dataset while maintaining stable generalization under a leakage-free evaluation protocol. Anchor explanations generated concise, high-precision IF–THEN rules that explained individual predictions, whereas complementary feature-importance analyses identified consistent financial drivers across models. Causal inference showed that Net Profit Margin Change, Sales Growth, EBIT, and Asset Turnover exert positive causal effects on the probability of future earnings growth, while Inventory to Total Assets and Cash Flow to Net Income exhibited negative causal effects. Placebo and refutation tests supported the robustness of the estimated treatment effects. TRuE-XAI integrates predictive modelling, explainable AI, visual analytics, and causal inference into a unified framework for transparent earnings-growth forecasting. By combining competitive predictive performance with interpretable decision rules and causally grounded insights, the framework provides a practical and trustworthy approach for financial decision support and demonstrates how explainable and causal machine learning can be applied in regulated financial environments.

## 1 Introduction

Forecasting corporate earnings plays a vital role in financial decision-making, benefiting a wide array of stakeholders. Financial analysts depend on earnings forecasts to develop reliable investment recommendations, while investors use them to identify stable, high-performing companies (Siladjaja and Jasman, 2024). Lenders evaluate earnings potential to assess credit risk and repayment capacity (Awad et al., 2023). Within organizations, earnings forecasts are critical for strategic planning, enabling management to allocate resources efficiently and pursue growth objectives. Consequently, the demand for accurate and transparent forecasting tools is substantial. These models often rely on indicators such as profitability ratios, cash flow performance, leverage, liquidity, and other accounting metrics. Although recent studies highlight the predictive value of alternative data sources such as textual sentiment, ESG scores, and macroeconomic indicators, this study deliberately focuses on audited financial-statement variables to establish a controlled and regulator-aligned baseline. The proposed TRuE-XAI framework is explicitly designed to be extensible to such alternative data modalities in future work. The current U.S. SEC(United States Securities and Exchange Commission) implementation demonstrates that trustworthy, causally grounded earnings-growth forecasting can be achieved using standardized regulatory data alone, thereby strengthening its applicability in compliance-sensitive financial environments.

### 1.1 Relevance of earnings forecasting

A natural question is why investors and institutions require earnings forecasts when firms already publish quarterly or annual reports. In practice, capital markets are forward-looking: prices, credit spreads and regulatory assessments depend on expectations of future profitability rather than past realizations. Forward-looking earnings estimates enable investors and lenders to anticipate turning points before they appear in official disclosures, rebalance portfolios, hedge exposures and adjust credit terms proactively. Directional forecasts (increase vs. decrease) are particularly useful for trading and risk management decisions, while forecasts of magnitude support valuation models, scenario analysis and capital planning. Moreover, earnings outcomes are influenced by non-financial drivers such as governance quality, managerial tone, macroeconomic and geopolitical sentiment, supply-chain conditions and ESG indicators. These factors are conceptually important but are not modeled explicitly in this study due to data availability and scope constraints; we instead focus on financial-statement variables as a first, controlled setting, with non-financial extensions identified as future work.

### 1.2 Research gap and study objective

Despite substantial progress in earnings forecasting, current approaches remain limited in several ways. Classical statistical and econometric models typically rely on linearity and stationarity assumptions, which restrict their ability to capture nonlinear interactions and regime changes in financial data. Modern machine learning (ML) models–such as Random Forest, gradient boosting and deep networks–address these limitations and often achieve superior predictive performance, but they frequently operate as opaque “black boxes”, offering limited insight into why a particular forecast is issued. Existing explainable AI (XAI) tools, including SHAP and LIME, provide post-hoc feature attributions but do not, by themselves, distinguish between causal drivers and spurious correlations, which is problematic in regulated, high-stakes environments where accountability is essential. Furthermore, most prior work treats prediction, explainability and causal reasoning as separate tasks: causal inference is rarely integrated into the same pipeline as predictive optimization and visual analytics, and comparisons to standard XAI tools are often qualitative rather than systematic.

To address these limitations, this study proposes TRuE-XAI (Transparent, Rule-based and Explainable AI)-a holistic framework that combines ensemble learning, structural causal modeling and interpretable rule generation for earnings-growth prediction. The goal is not only to classify the direction of earnings changes, but also to quantify how changes in key financial variables and ratios affect the probability of earnings growth, thereby bridging the gap between directional market signals and economically meaningful effect sizes. This research makes four main contributions to the field of explainable and trustworthy financial forecasting.

1. **Integrated framework**. We introduce TRuE-XAI, a unified architecture that combines hyperparameter-optimized ensemble learning, imbalance-aware sampling, causal inference and human-interpretable rule-based explanations to ensure both predictive performance and transparency in earnings-growth prediction.
2. **Causal explainability**. Unlike conventional XAI approaches such as SHAP and LIME, which focus on associative feature attributions, TRuE-XAI embeds structural causal models (SCMs) and counterfactual reasoning (via DoWhy and EconML) to uncover genuine cause-effect relationships between financial indicators and earnings-growth outcomes.
3. **Predictive performance**. Through automated hyperparameter optimisation (Optuna and Hyperopt) and advanced class-rebalancing techniques (SMOTE, ADASYN, TomekLinks, RENN), the framework achieves competitive and stable F1-scores (0.35-0.47 on real SEC data) on a severely imbalanced earnings dataset, with overfitting monitored through temporal cross-validation and train-test gap analysis.
4. **Comprehensive evaluation and visualization**. TRuE-XAI provides a reproducible experimental pipeline, combining quantitative benchmarks, robustness and refutation tests, and interactive SilVA visual analytics to support qualitative inspection of Random Forest decision paths and feature hierarchies. This allows practitioners to cross-check local Anchor rules, global feature importance and causal estimates within a coherent visual and analytical environment.

Collectively, these contributions position TRuE-XAI as a scientifically grounded and practically applicable approach for developing trustworthy AI systems in finance, with potential extensions to other regulated domains such as healthcare, auditing and law. To ensure external validity, all primary experiments were conducted on real-world SEC filings; simulation is included only for controlled methodological verification in the Appendix.

### 1.3 Scientific importance

Beyond its empirical performance, the scientific significance of this work lies in advancing an emerging paradigm of *trustworthy, causally informed XAI* for financial modeling. While much of the existing literature focuses either on predictive accuracy or on post-hoc explanations, TRuE-XAI unifies three complementary research dimensions–predictive optimization, causal reasoning and human-interpretable explanation–within a single, integrated framework. This synthesis transforms earnings forecasting from a purely correlational exercise into a causally informed, transparent and reproducible process. By embedding SCMs and treatment-effect estimation into explainable ensemble learning, the framework illuminates how financial variables interact to drive earnings growth, rather than merely ranking them by association strength. This methodological integration responds directly to recent calls for AI systems that are not only accurate but also causally robust, auditable and aligned with domain knowledge.

Historically, statistical models have been employed to analyze earnings behavior. Early work by Ball and Watts (1972) examined whether earnings followed a random walk or exhibited more complex temporal dynamics, finding that deviations from the random-walk benchmark did exist. Freeman et al. (1982) demonstrated that incorporating additional explanatory variables–such as book value–alongside historical earnings improved predictive accuracy. Hou et al. (2012) proposed a cross-sectional model-based approach to estimate the implied cost of capital, outperforming analyst forecasts by offering broader coverage and reduced bias. Gerakos and Gramacy (2013) compared various regression techniques and showed that even basic linear models, when combined with lagged income variables, can yield strong predictive results. Hybrid approaches that blend analyst judgment with model-based adjustments have also been explored; for example, Azevedo et al. (2021) balanced human forecasts with systematic updates derived from recent market behavior. However, the growing complexity and volume of financial reporting have exposed the limitations of traditional econometric methods, motivating the adoption of machine learning as a scalable, data-driven alternative.

Recent advances in ML-based earnings prediction have demonstrated considerable promise. Ishibashi et al. (2016) applied feature-selection algorithms–including correlation-based methods and decision trees–to identify influential predictors from financial statement data. Anand et al. (2019) used Random Forests to anticipate directional changes in profitability metrics such as ROA and FCF, achieving significantly better accuracy than random-walk baselines. Nguyen (2020) evaluated gradient-boosted regression trees and found mixed evidence relative to human forecasts. Hunt et al. (2022) compared various classifiers and concluded that Random Forests not only offered superior predictive accuracy but also enabled profitable trading strategies. Chen et al. (2022) demonstrated that ML models applied to high-dimensional datasets yield notable gains in forecasting one-year-ahead earnings directions. Other studies, including Belesis et al. (2023) and Hess et al. (2024), emphasized the predictive advantage of cash-flow metrics over accrual-based measures and showcased the long-term forecasting power of flexible ML pipelines.

Despite these advances, important challenges remain–particularly around interpretability and causal validity. Most high-performing ML models function as black boxes, offering little transparency into how or why specific predictions are made. This opacity is problematic in regulated financial environments, where decisions must be explainable, auditable and robust to shifts in market conditions. Standard feature-importance metrics provide partial insight, but they do not reveal whether features *cause* changes in earnings or simply co-move with them, nor do they easily support counterfactual reasoning (e.g., “What happens to the likelihood of earnings growth if profit margins increase while inventory intensity decreases?”).

This limitation has catalyzed interest in causal inference techniques within ML research. Frameworks such as structural equation modeling, graphical causal models and potential-outcomes theory now support systematic analysis of cause-effect relationships, but they remain underutilized in earnings forecasting. In domains such as finance, healthcare and public policy, causality-aware models are essential for actionable insights: identifying whether a change in Net Profit Margin leads to earnings growth, rather than merely coinciding with it, is crucial for designing effective interventions and policies. At the same time, explainable AI has matured, with tools like SHAP and LIME offering model-agnostic attributions; however, these methods generally operate at the level of association and do not guarantee causal interpretability. The gap between high-performing black-box models and scientifically grounded causal explanations motivates the hybrid approach developed in this paper.

TRuE-XAI addresses this gap by integrating causal inference, explainability and predictive optimization in a single workflow. DoWhy is used to construct and identify structural causal models for selected financial treatments and earnings outcomes; EconML's Double Machine Learning estimators quantify heterogeneous treatment effects; Anchor explanations provide sparse, high-precision rules for individual predictions; and SilVA visual analytics expose decision paths within Random Forest ensembles. Together, these components ensure that the model is not only accurate, but also interpretable and causally informative.

The study aims to address the following research questions:

- **RQ1**: How can machine learning models be optimized to forecast earnings growth using financial statement data?
- **RQ2**: Which financial indicators have the most significant influence on earnings increases or decreases?
- **RQ3**: How can explainable AI approaches foster trust in black-box model predictions?
- **RQ4**: In what ways can causal inference be combined with machine learning to uncover the cause-and-effect dynamics behind financial variables and future earnings growth?

By answering these questions, this research advances the field of explainable and trustworthy AI in financial modeling. The rest of this paper is organized as follows: Section II reviews related work; Section III describes the proposed methodology; Section IV outlines the experimental setup and results; Section V discusses key findings and implications; and Section VI concludes with final remarks.

## 2 Related work

### 2.1 Earning growth indicators

Forecasting future earnings relies heavily on understanding a firm's financial position, performance trajectory, and structural determinants of profitability. Early studies such as Ou and Penman (1989) demonstrated that financial statement variables contain predictive signals about upcoming changes in earnings. Their framework used accounting ratios to classify whether a firm's earnings would rise or fall in the subsequent year. Ishibashi et al. (2016) further extended this stream by modeling earnings-per-share (EPS) (Equation 1) dynamics using drift-adjusted metrics to estimate the relative likelihood of earnings changes.


$$\begin{array}{l} \begin{array}{l} {\mathrm{Pr}}_{i}\left(t+1\right)\\ \;=\{\begin{array}{cc} 0 & \left(e.p.s_{i}\left(t+1\right)-e.p.s_{i}\left(t\right)-drift_{i}\left(t+1\right)<0\right)\\ 1 & \left(e.p.s_{i}\left(t+1\right)-e.p.s_{i}\left(t\right)-drift_{i}\left(t+1\right)>0\right), \end{array} \end{array} \end{array}$$ (1)

The term *drift*_*i*_(*t* + 1) refers to the average change in earnings per share (EPS) over the four years beginning with fiscal year (*t* + 1). If *Pr*_*i*_(*t* + 1) represents the company's earnings growth, then a value of *Pr*_*i*_(*t* + 1) = 0 indicates a decrease in earnings.

While such approaches provide foundational insights, they rely on linear assumptions and do not capture nonlinear interactions or feature interdependencies. More recent studies focus on predicting financial distress and earnings outcomes using accounting ratios, credit spreads, or macroeconomic indicators. However, these models often aggregate historical patterns without addressing temporal dependencies, structural causality, or explanation grounded in decision reasoning. As non-financial signals (e.g., governance, sentiment) also influence earnings but are often absent in traditional predictor-based frameworks.

### 2.2 Explainable AI in financial decision-making

Explainable Artificial Intelligence (XAI) has emerged as an essential dimension of responsible financial analytics, particularly where model outputs affect investment decisions, creditworthiness assessments, and regulatory compliance. XAI frameworks aim to enhance transparency by enabling users to understand the rationale behind model predictions, identify reliability bounds, and evaluate the robustness of decisions under different conditions (Miller, 2019; Ortigossa et al., 2024).

In financial services, AI has improved performance in credit scoring, risk assessment, portfolio management, and fraud detection. However, the opacity of high-performing ML models raises concerns related to fairness, accountability, and interpretability. Regulators increasingly require that financial institutions justify algorithmic decisions, especially when automated systems affect access to credit, pricing, or capital allocation (Kute et al., 2021).

To bridge this gap, recent research has applied model-agnostic XAI tools such as SHAP and LIME to financial applications. Medianovskyi et al. (2022), Aruleba and Sun (2024), De Lange et al. (2022), Tran et al. (2022), and Nallakaruppan et al. (2024) demonstrated how SHAP-based explanations can improve trust in credit risk and distress-prediction models. These studies consistently show that interpretability strengthens model acceptance. However, SHAP and LIME provide correlation-based explanations rather than causal ones. They do not reveal how variables interact under counterfactual changes and cannot confirm whether a feature genuinely influences earnings outcomes. This limitation motivates the integration of causal inference within explainable predictive systems.

### 2.3 SHAP-based explainability in financial modeling

SHAP has become one of the most widely used interpretability methods due to its solid theoretical basis in cooperative game theory. Studies applying SHAP to financial datasets—such as SME distress prediction (Medianovskyi et al., 2022), credit scoring (Aruleba and Sun, 2024; De Lange et al., 2022), loan default prediction, and corporate distress forecasting Tran et al. (2022); Nallakaruppan et al. (2024) –demonstrate its effectiveness in ranking influential features and offering global and local explanations. SHAP decomposes predictions into additive Shapley values, allowing consistent attribution of each feature's marginal contribution.

Similarly, Tran et al. (2022) demonstrated that SHAP-enhanced Random Forest and XGBoost models yielded strong performance in predicting financial distress among Vietnamese public firms, with critical drivers such as long-term debt to equity, enterprise value to revenue, and diluted earnings per share. Nallakaruppan et al. (2024) integrated SHAP, LIME, and partial dependence plots within a Random Forest framework, achieving 0.998 accuracy, 0.998 sensitivity, and 0.997 specificity on a loan prediction dataset, thereby providing highly interpretable outputs for credit decisions.

Underlying all these applications is the theoretical foundation introduced by Lundberg and Lee (2017), who formalized SHAP as a unified framework for interpreting complex machine learning models. SHAP assigns each feature a Shapley value ∅_*i*_, reflecting its marginal contribution to the model's prediction. The Shapley value for feature *i* is defined as the average of its marginal contributions across all possible subsets *S* ⊆ *N*\{*i*}, where *N* is the set of all features and *n* = |*N*|, as given in Equation 2.


$$\begin{array}{l} \emptyset_{i}=\frac{1}{n!}\underset{S\in N-\left\{i\right\}}{\sum }|S|!\left(n-1-|S|\right)!\left[f\left(S\cup \left\{i\right\}\right)-f\left(S\right)\right] \end{array}$$ (2)

Here, *f*(*S*) denotes the model's prediction based only on subset *S*, ensuring that each feature's contribution is fairly distributed. For a specific prediction instance *x*, SHAP approximates the original model *f*(*x*) using a linear surrogate model *g*(x^*^), described in Equation 3.


$$\begin{array}{l} f\left(x\right)=g\left(x^{*}\right)=\emptyset_{0}+\overset{M}{\underset{i=1}{\sum }}\emptyset_{i}x_{i} \end{array}$$ (3)

In this formulation, ∅_0_ is the base value (i.e., expected model output without any feature information), *M* is the total number of simplified features, and *x*_*i*_ represents the input for feature *i*. This linear explanation model ensures local accuracy and additive feature attribution, making SHAP especially effective for both global model insights and local prediction explanations.

Despite its popularity, SHAP faces practical limitations relevant to this study:

1. Causality: SHAP does not differentiate between correlation and causation.
2. Feature interactions: SHAP may overemphasize correlated features.
3. Model reliance: SHAP approximations depend on assumptions that may not fully reflect complex financial interactions.
4. Explainability gap: SHAP values do not produce human-readable IF-THEN rules, making explanations less accessible for auditors, regulators, or financial managers.

These limitations directly align with concerns regarding the overreliance on SHAP/LIME in existing research and the need for more robust interpretability.

### 2.4 Visual interpretability of Random Forest models using SilVA

As Jamnal (2023) proposed, SilVA (Structured Interpretability for Latent Visual Analytics) presents a visual analytics approach for interpreting Random Forest models. It provides hierarchical and interactive visualizations—such as feature-ranking comparisons, icicle plots, and decision-flow summaries—to expose dominant decision paths and model logic. The system integrates multiple feature importance metrics and visualizes structure across trees, enabling users to explore root-to-leaf patterns.

Within the SilVA system, multiple feature importance measures—including root node frequency, split frequency, Gini-based impurity reduction, permutation importance, ANOVA-based scoring and SHAP-based importance—were integrated and visualized through coordinated views. Ranking stability was assessed using Kendall's(τ) correlation coefficient and dense ranking. To facilitate transparency, icicle plots were employed to represent tree structures, showing feature splits from root to leaf. A prediction popularity module was implemented to visualize class label voting distributions across trees, and a Sankey-style Decision Flow Summary (DFS) diagram was introduced to summarize common feature pathways. The framework was applied to two benchmark datasets—UCI's Wine Quality and Kaggle's Letter Recognition—where it was reported that top-ranking features such as *alcohol, residual sugar, xybar*, and *ybar* could be consistently identified across multiple model instances.

Although SilVA faces several limitations: (a) Visualizing individual trees does not always generalize to ensemble-level behavior. (b) Scalability and cognitive load can overwhelm non-technical users. (c) Interpretability effectiveness is not quantitatively validated for complex, imbalanced financial datasets.

In TRuE-XAI, SilVA is therefore used as a supplementary visualization tool, not the primary explanation mechanism. Its purpose is to support exploratory analysis rather than serve as the central interpretability technique.

### 2.5 Causal discovery and inference

**Figure 1:**
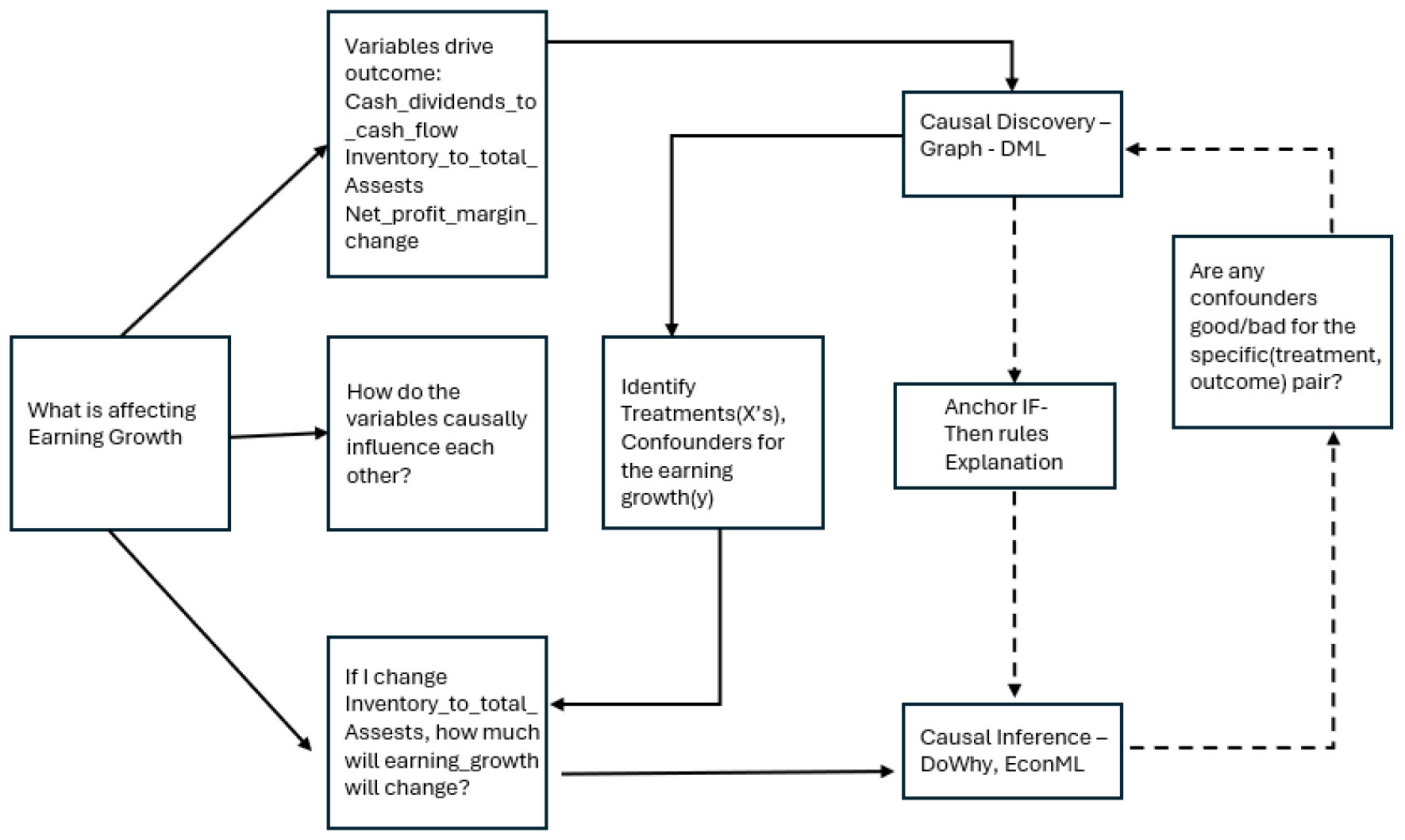
Flowchart depicting methods for causal question creation.

Causal inference methods have increasingly been applied in machine learning to distinguish genuine cause-effect mechanisms from spurious correlations. Tools such as DoWhy (Sharma and Kiciman, 2020) and EconML (Keith Battocchi, 2019) operationalize causal reasoning by enabling the identification, estimation, and refutation of treatment effects. These methods allow researchers to model interventions (e.g., increasing net profit margin) and assess their effect on outcomes (e.g., likelihood of earnings growth). Figure 1, represents flowchart for the casual question creation.

Applications of DoWhy in fields such as transportation safety (Liu et al., 2022), renewable energy forecasting (Wang et al., 2023), environmental sustainability (Cabije et al., 2024), and healthcare risk analysis (Noh and Kim, 2024) show its versatility in uncovering actionable insights. EconML extends causal estimation to heterogeneous treatment effects using advanced meta-learners, double ML, and orthogonal forests (Chernozhukov et al., 2018).

DoWhy breaks down causal analysis into four main steps:

- Modeling: A causal graph is first constructed to encode assumptions about relationships between variables. The graph need not be exhaustive, but it must reflect known influences. Any missing links are treated as potential confounders (Sharma and Kiciman, 2020).
- Identification: Given the causal graph, DoWhy determines whether the target causal effect can be identified from the data. In this study, structural causal models (SCMs) were defined where financial indicators—such as Sales Growth and Net Margin—served as potential treatments influencing the binary outcome of earnings growth. Identification used the back-door criterion, adjusting for confounders such as Operating Margin, Asset Turnover, Receivable Turnover and Return On Equity, Cash Flow-to-Net Income. Treatment effects were then estimated via linear regression and validated using placebo refutation and random confounder injection. For example, increasing Net Margin causally increased the probability of earnings growth by 19.3 percentage points (*p* < 0.01), confirming its importance as a high-leverage driver Sharma and Kiciman (2020); Pearl (2009).
- Estimation: After identification, DoWhy applies statistical estimators to quantify causal effects. Supported methods include: Treatment-assignment approaches (matching, stratification, inverse probability weighting), Outcome-based models (linear and generalized linear regression), Instrumental-variable techniques (binary IV estimation, two-stage least squares), and Mediation analysis via two-stage regression. DoWhy also provides confidence intervals and significance testing using permutation procedures.
- Refutation: The robustness of estimated effects is tested through multiple refutation strategies (Sharma and Kiciman, 2020), including: Adding random confounders, Applying placebo treatments, Evaluating synthetic outcomes from known data-generating processes, Sensitivity analysis for unmeasured confounding, and Validation across data subsets or bootstrap samples.

In addition to DoWhy, the EconML library (Keith Battocchi, 2019) offers advanced tools for estimating heterogeneous treatment effects in high-dimensional settings. It combines machine learning with causal inference to model how treatment effects vary across individuals or subgroups. Key estimators include

- Double Machine Learning (DML) for controlling high-dimensional confounders (Chernozhukov et al., 2018).
- Meta-learners(T-, S-, and X-learners) for flexible treatment-effect estimation from observational data.
- Orthogonal Random Forests, which capture nonlinearities and complex interactions between features and treatment effects.

Despite significant progress, causal inference remains underutilized in earnings growth forecasting, especially when integrated with predictive ML and XAI. Existing studies seldom combine causal graphs, treatment-effect estimation, and post-hoc explanations within a unified predictive pipeline.

### 2.6 Anchor: a high-precision, model-agnostic explanation method

Anchor explanations provide model-agnostic, high-precision IF-THEN rules that guarantee locally stable predictions under perturbation. Anchors generate sufficient conditions under which a model's output is unlikely to change–an interpretable mechanism particularly suitable for financial decision-making, where stakeholders require transparent and audit-ready justification. Each rule, referred to as an “anchor,” identifies specific feature value ranges under which the prediction is stable with high precision. For example, a rule like “Net Profit Margin Change ≤ -0.5 and Gross Profit ≤ -0.2” would consistently lead to a prediction of earnings decline across similarly perturbed samples. During our implementation, rules with minimum precision thresholds of 95% were retained. This ensured that the explanations not only captured local fidelity but also provided actionable insights into dominant financial indicators driving prediction outcomes. Compared with SHAP and LIME, Anchors offer: (a) Human-readable rule sets, (b) High-precision thresholds, (c) Consistency across local perturbations, and (d) Stronger actionable insights.

A key advantage of Anchor is its dual emphasis on coverage and precision. Coverage represents how broadly a rule applies to instances in the local neighborhood, while precision quantifies the likelihood that the model's prediction remains unchanged within this subset. Anchors are only considered valid if they satisfy a user-defined precision threshold τ (e.g., τ = 0.95), ensuring robust and trustworthy explanations. Formally, an anchor *A* for a data instance *x* must satisfy the condition presented in Equation 4:


$$\begin{array}{l} {𝔼}_{D_{x}\left(z\cdot |A\right)}\left[1\left\{\hat{f}\left(x\right)=\hat{f}\left(z\right)\right\}\right]\geq \tau \text{ subject to }A\left(x\right)=1 \end{array}$$ (4)

Here:

- *x* is the data point being explained,
- *A* is a set of conditions (i.e., the anchor),
- $\hat{f}$ is the black-box model,
- *D*_*x*_(*z*·|*A*) is the distribution of perturbed instances near *x* satisfying anchor *A*,
- τ ∈ [0, 1] is the required precision threshold.

Furthermore, precision serves as a core metric for evaluating the reliability of an anchor rule. It is defined as the conditional probability that the model's prediction remains the same for any perturbed instance *z* satisfying the anchor conditions expressed in Equation 5.


$$\begin{array}{l} \text{Precision}\left(A\right)=\mathbb{P}\left(\hat{f}\left(z\right)=\hat{f}\left(x\right)|A\left(z\right)=1\right) \end{array}$$ (5)

High precision (close to 1.0) indicates that whenever the anchor conditions hold, the model consistently outputs the same prediction as for the original instance *x*. Anchor algorithms typically impose a minimum precision threshold (e.g., τ = 0.95) to ensure that generated explanations remain consistent and actionable across perturbations. Because Anchor does not depend on the internal structure of the model, it is highly generalizable. However, Anchors are rarely applied to financial earnings forecasting, creating a research gap directly addressed by TRuE-XAI.

### 2.7 Data balancing techniques

Class imbalance is common in financial datasets and it distorts learning. Studies commonly use SMOTE, ADASYN, Tomek Links, and RENN to manage imbalance and improve classifier robustness. Each balancing method was encapsulated within a pipeline that also included feature standardization and classification, enabling consistent comparative evaluation across XGBoost, Random Forest, and LightGBM models. Classification performance was assessed using F1-score and accuracy metrics, ensuring sensitivity to false positives and false negatives. The empirical results demonstrated that hybrid pipelines incorporating ADASYN and SMOTE significantly improved recall and F1-score, confirming the importance of tailored resampling strategies in achieving robust predictive performance in imbalanced financial datasets.

However, resampling may not always benefit ensemble methods, and the need for class balancing depending on the problem setup. To address both concerns, TRuE-XAI evaluates multiple balancing strategies empirically and reports their influence on model performance, avoiding assumptions about their universal effectiveness.

### 2.8 Hyperparameter optimization

Hyperparameter optimization frameworks such as Optuna and Hyperopt enable systematic improvements in ML models through probabilistic search strategies. While widely used in ML research, their application to explainable earnings forecasting remains limited. Integrating these tools supports for stronger technical grounding and concerns about methodological clarity. To enhance model generalization and predictive accuracy, automated hyperparameter optimization frameworks–Optuna and Hyperopt were integrated into the training workflow. For XGBoost and Random Forest, Optuna's Tree-structured Parzen Estimator (TPE) sampler efficiently navigated the hyperparameter space, optimizing parameters such as n_estimators, max_depth, and learning_rate. In parallel, LightGBM was fine-tuned using Hyperopt, which employed probabilistic search over discrete and continuous spaces, including num_leaves and learning_rate. Each optimization trial involved 3-fold cross-validation using F1-score as the evaluation metric, ensuring that hyperparameter tuning was both performance-driven and statistically grounded. This dual-framework strategy enabled a fair comparison across models while leveraging the strengths of both optimization libraries. Ultimately, the systematic tuning process contributed to significant performance improvements, particularly when combined with resampling techniques, thereby validating the necessity of hyperparameter optimization in complex, imbalanced classification tasks. Optuna is a popular open source library that simplifies the process of hyperparameter tuning in machine learning projects, while hyperopt strength lies in its adaptive resource allocation mechanism, which initially assigns a small amount of resource to each setup and gradually increases the allocation for those showing promise.

## 3 Proposed methodology

### 3.1 Framework overview and technical integration

The TRuE-XAI framework is implemented as a multi-stage pipeline that integrates data preprocessing, ensemble learning, causal inference, and explainable analytics into a unified decision-support architecture. Its structure is designed to ensure that model predictions are not only accurate, but also causally grounded and interpretable for financial end-users.

The framework consists of four tightly connected components. First, financial and macroeconomic indicators are cleaned, standardized, and balanced using adaptive resampling methods to correct class imbalance. This step clarifies why balanced directional forecasting is required and addresses concerns about motivation for sampling in imbalanced datasets. Second, predictive optimization is performed using hyperparameter-tuned ensemble learners—Random Forest, XGBoost, and LightGBM—which capture nonlinear relationships among financial variables. Third, structural causal models (SCMs) implemented through DoWhy and EconML enable the identification, estimation, and validation of treatment effects, thereby distinguishing causal drivers from spurious correlations. This addresses a clearer justification of SCMs by demonstrating how SCMs enable intervention-based reasoning rather than correlation analysis alone. Finally, interpretability is delivered through Anchor-based rule extraction and SilVA visual analytics, which provide local explanation rules and structural insights into ensemble decision paths. Together, these components operate sequentially and inform one another, ensuring that predictive patterns are supported by causal reasoning and transparent explanations.

**Figure 2:**
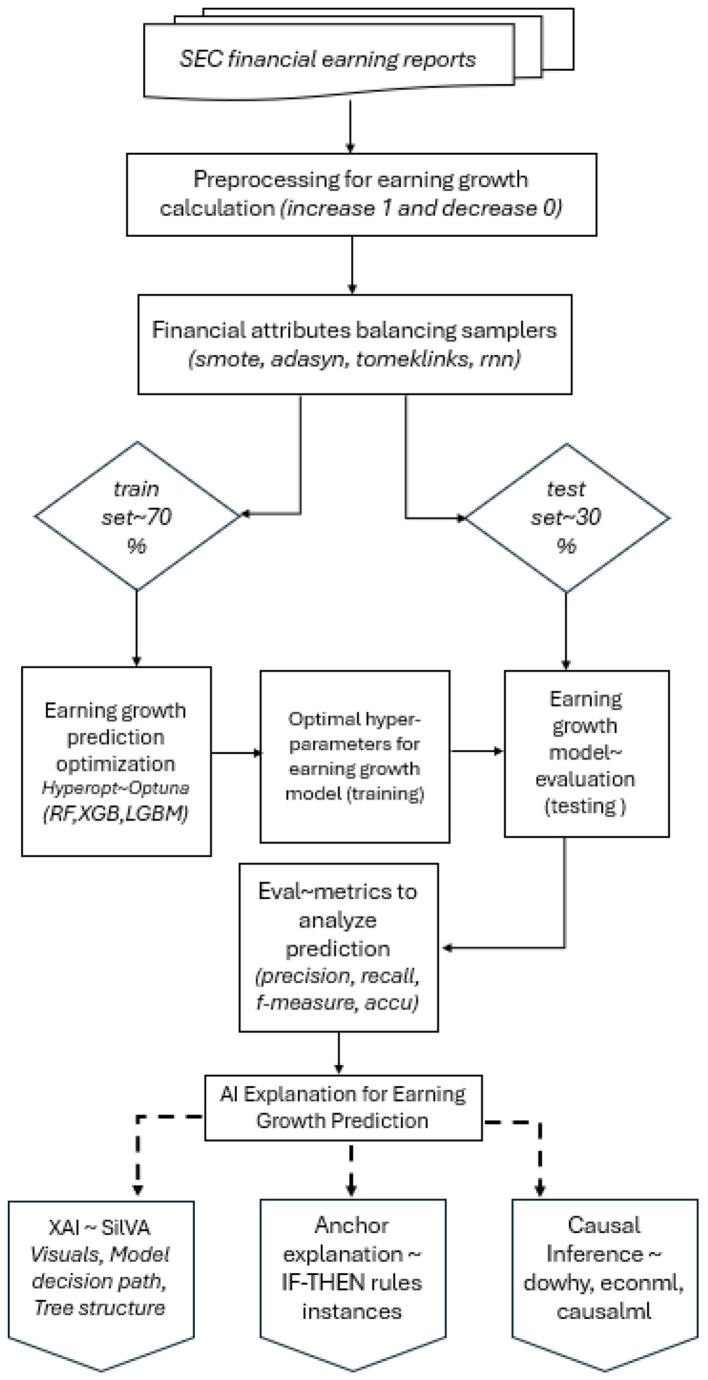
Proposed TRuE-XAI framework of earnings growth predictions through optimized Random Forest with causal and explainable AI.

Figure 2 illustrates the complete TRuE-XAI pipeline for earnings-growth forecasting. To address class imbalance and enhance predictive performance, automated hyperparameter optimization (via Optuna and Hyperband) is combined with resampling techniques including SMOTE, ADASYN, TomekLinks, and RENN. All components are embedded within standardized pipelines and applied consistently across ensemble models. Model selection is guided by F1-score-based cross-validation, ensuring robustness in imbalanced settings. A major methodological enhancement in this work is the explicit positioning of TRuE-XAI as a scientific contribution, not merely an application, by explaining how its integrated architecture advances trustworthy AI through the combined use of optimized ML, SCM-based causal inference, and interpretability tools.

Beyond predictive accuracy, TRuE-XAI emphasizes interpretability and analytical depth. Anchor explanations provide precise, model-agnostic rules that clarify local decision behavior and highlight influential financial indicators. Causal inference modules further uncover the underlying mechanisms driving earnings growth by estimating treatment effects and counterfactual responses. SilVA analytics complement these insights by visualizing the internal structure of ensemble models, revealing decision hierarchies and feature interaction pathways. This combination of explainability, causal reasoning, and visual interpretability supports trustworthy forecasting and enhances user confidence in model outputs.

### 3.2 Advancement of earning growth forecasting through optuna and hyperopt optimization

To improve predictive accuracy in earnings growth forecasting, the proposed approach is structured around two core components, summarized in Algorithm 1: data balancing and automated hyperparameter optimization. These steps are essential for mitigating the challenges posed by class imbalance and ensuring strong generalization performance in financial classification models. By systematically addressing both data-quality and model-configuration issues, the TRuE-XAI framework enhances robustness and stability in forecasting complex earnings outcomes. In particular, this dual strategy ensures that the models do not simply fit historical patterns but remain reliable when confronted with new firms or changing market conditions, directly responding to concerns about overfitting and practical usefulness.

Algorithm 1 Leakage-free earnings growth prediction with temporal split, group-aware CV, training-only balancing, and hyperparameter optimization. 
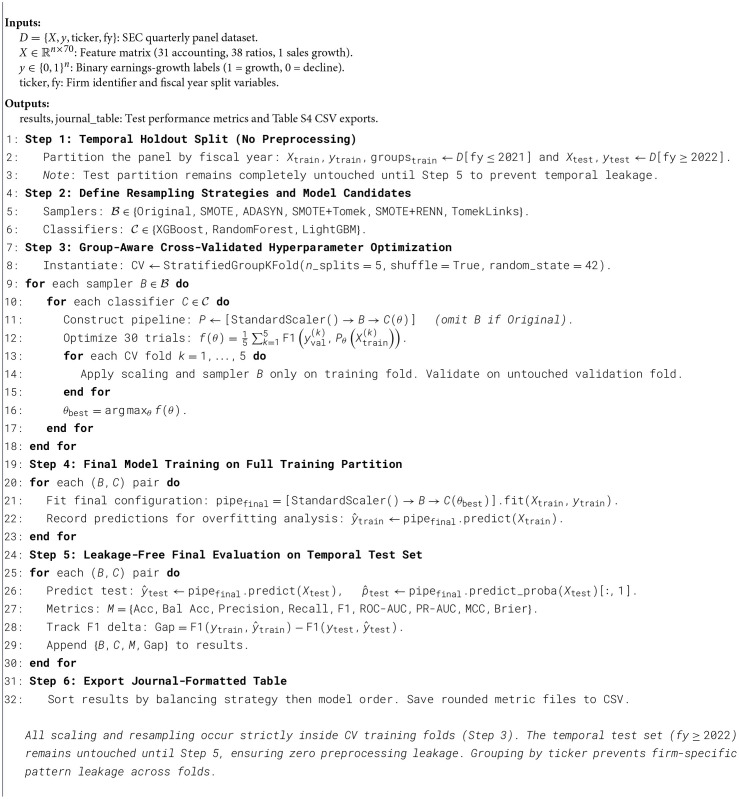

To enhance predictive accuracy and mitigate the challenges of skewed class distributions, TRuE-XAI integrates resampling-based data balancing with Optuna- and Hyperopt-driven hyperparameter tuning, as detailed in Algorithm 1. These two components work jointly to ensure reliable and generalizable performance in earnings-growth classification tasks. By optimizing model parameters while simultaneously correcting for distributional imbalance, the framework strengthens its resilience and improves the stability of earnings-growth predictions across diverse financial environments. This clarifies the methodological role of optimization and resampling beyond mere performance enhancement and positions them as core elements of a scientifically grounded forecasting pipeline, addressing concerns about methodological significance.

#### 3.2.1 Imbalanced data treatment using SMOTE, ADASYN, Tomek Links, and RENN

In earnings growth prediction, imbalanced data can severely hinder the learning capacity of classifiers by biasing them toward majority class labels. To alleviate this, the study integrates a suite of resampling techniques comprising both oversampling and undersampling methods into a unified pipeline. Oversampling is performed using SMOTE and ADASYN, which synthetically generate new instances of the minority class to promote class parity and increased exposure to underrepresented patterns. In contrast, Tomek Links (Devi et al., 2017) and RENN (Repeated Edited Nearest Neighbors) are employed as undersampling techniques to remove overlapping (Guan et al., 2009) or noisy examples from the majority class, thereby refining decision boundaries and reducing ambiguity in the feature space. This combination of oversampling and undersampling explicitly addresses the risk that a single resampling method might either oversimplify the class boundary or introduce unrealistic synthetic examples.

These methods are encapsulated within scikit-learn-compatible pipelines, which also include feature standardization and classifier modules to ensure consistent preprocessing and evaluation. Comparative experiments across XGBoost, Random Forest, and LightGBM demonstrate that integrating ADASYN and SMOTE substantially enhances F1-score, precision, recall, and accuracy metrics, highlighting the efficacy of adaptive resampling for addressing imbalanced financial datasets. At the same time, we acknowledge that oversampling may increase the risk of overfitting in regions with few original samples; therefore, performance is always assessed using cross-validation and multiple metrics rather than relying on accuracy alone, in line with concerns regarding apparently high scores.

#### 3.2.2 Exploiting optuna and hyperopt for hyperparameter optimization

To maximize model generalization and predictive power, automated hyperparameter optimization frameworks were incorporated into the training pipeline. Optuna was employed for optimizing XGBoost and Random Forest models using its Tree-structured Parzen Estimator (TPE) sampler, effectively searching parameter spaces such as n_estimators, max_depth, and learning_rate (Shekhar et al., 2021). For LightGBM, the Hyperopt framework was used, leveraging probabilistic sampling strategies to optimize num_leaves, n_estimators, and learning_rate (Probst et al., 2019). This systematic search replaces ad-hoc parameter selection and ensures that performance gains are attributable to well-defined optimization rather than manual trial-and-error, thereby strengthening the technical soundness of the methodology.

All optimization routines used 3-fold cross-validation, with F1-score, precision, recall and accuracy as the target metrics, ensuring robust and balanced evaluation under imbalanced class conditions. F1-score was chosen as the primary objective to balance precision and recall, directly addressing concerns that accuracy alone can be misleading when class distributions are highly skewed. Each optimization process was embedded within an imbalanced-learn pipeline that included both the sampler and a StandardScaler, preserving consistency across evaluation scenarios and preventing information leakage between training and validation folds. The best-performing hyperparameter sets were logged for each model-balancing configuration, ensuring reproducibility (Putatunda and Rama, 2018). This explicit logging and pipeline-based design also facilitates independent replication and extension of the experiments, aligning with requests for improved transparency and reproducibility. The combination of hyperparameter tuning and tailored balancing significantly enhanced model performance, validating the integration of Optuna and Hyperopt as essential tools in financial machine learning pipelines. Moreover, the resulting well-regularized models provide a stable foundation for the downstream causal and explainable AI components of TRuE-XAI, ensuring that subsequent treatment-effect estimation and interpretation are based on robust predictive structures.

### 3.3 Explainable AI for future earnings prediction

To enhance model transparency in predicting future earnings, the TRuE-XAI framework incorporates multiple feature-importance strategies and local rule-based explanations, as presented in Algorithm 2. We evaluate six distinct importance extraction methods: (i) root count-based importance, which counts how frequently features appear at the root node of trees; (ii) feature split-based importance, measuring split frequency across the ensemble; (iii) variance impurity-based importance, derived from reductions in Gini impurity; (iv) permutation importance, which assesses performance degradation when features are randomly shuffled; and (v) ANOVA-based importance, which uses ANOVA F-tests to score each feature's relevance to the target variable and (vi) SHAP-based importance, which Quantifies feature impact on model predictions using game-theoretic Shapley values. These techniques offer both global and model-specific perspectives on feature relevance. Using multiple, complementary importance measures also allows us to assess ranking stability across methods, reducing the risk that conclusions depend on a single metric and addressing concerns about robustness in interpretability.

Algorithm 2 Explainable earnings prediction using feature importance and anchor rules. 
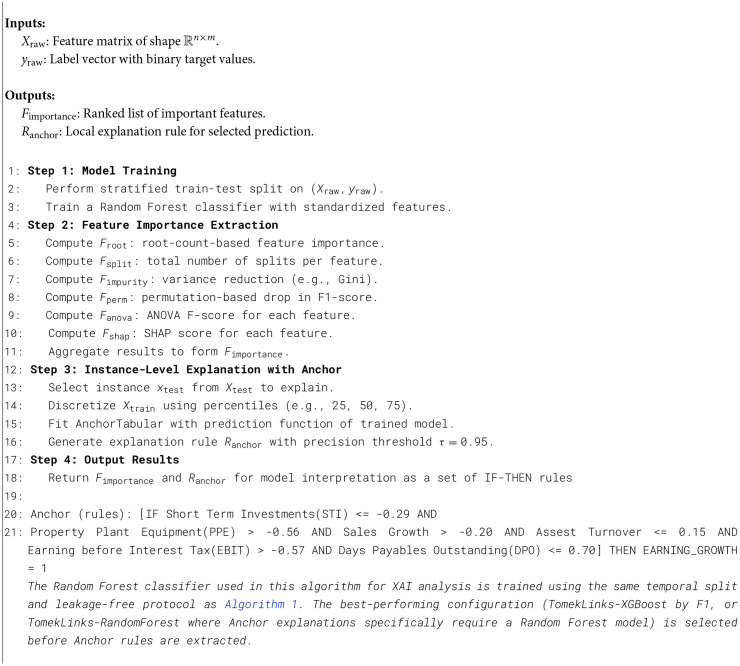

In addition, this research applies the Anchor algorithm to produce local, human-interpretable IF-THEN rules for individual predictions. Anchors define decision boundaries that lock predictions to a set of high-precision, easily understandable rules, significantly enhancing interpretability for end-users. The Anchor Tabular explainer is applied to Random Forest outputs, generating rules conditioned on discretized features. For each explanation, we report both precision and coverage, ensuring that the rules are not only locally faithful but also applicable to a meaningful subset of observations, as suggested in the review feedback. This hybrid approach ensures that feature-level insights and instance-level decisions are both interpretable, aligning with regulatory and business transparency requirements in financial earnings-growth forecasting. In the discussion section, we further compare Anchor-based rules conceptually with SHAP- and LIME-style attributions, clarifying the added value of rule-based explanations in terms of logical clarity and regulatory usability.

### 3.4 Causal discovery of factors influencing earnings growth using DoWhy and double machine learning

To complement predictive modeling with scientifically grounded explanation, TRuE-XAI incorporates a causal inference layer designed to estimate the directional influence of selected accounting variables on future earnings growth. The causal workflow, summarized in Algorithm 3, combines structural causal modeling (SCM) using DoWhy with Double Machine Learning (DML) using EconML to estimate both average and heterogeneous treatment effects under realistic financial data conditions.

Algorithm 3 Causal effect estimation for earnings growth using DML and DoWhy. 
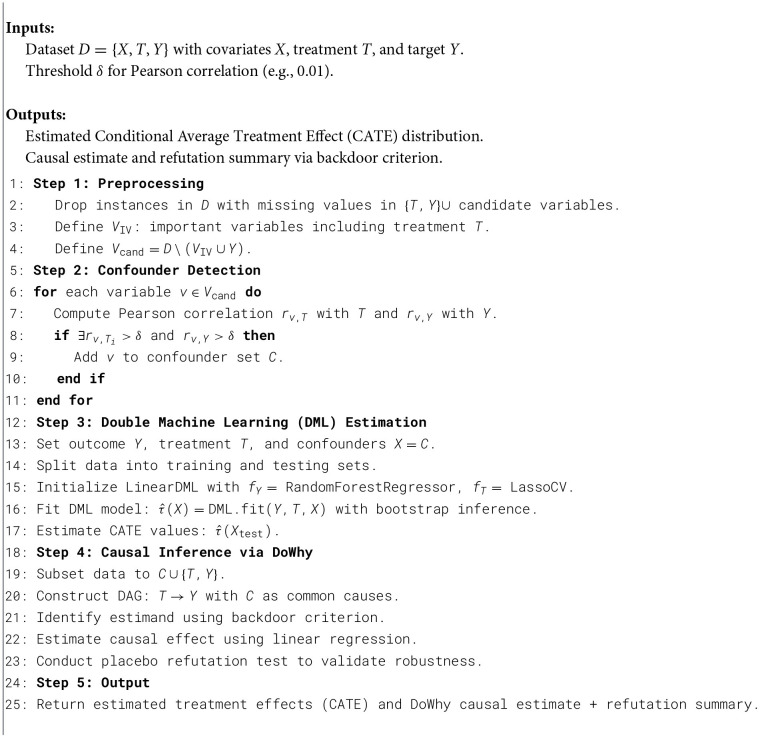

#### 3.4.1 Definition of outcome and candidate treatments

The outcome variable is defined as quarter-ahead earnings growth (binary directional label or continuous growth rate depending on the experiment). Candidate treatment variables were selected from financially interpretable indicators repeatedly highlighted in the forecasting literature and by feature-importance results, such as Sales Growth, Net Margin, Asset Turnover and Return on Equity.

#### 3.4.2 Confounder selection and justification

For each treatment, a set of confounders was defined based on accounting theory and causal plausibility. Specifically, confounders were required to influence both the treatment and the outcome, representing firm-level financial conditions that jointly affect profitability decisions and earnings dynamics (e.g., scale, liquidity, leverage, operating efficiency, and growth). In addition to domain reasoning, we applied a correlation screening rule to filter candidate covariates: only variables exhibiting statistically meaningful association with both the treatment and the outcome were retained as candidate confounders. This combined approach prevents purely data-driven inclusion while ensuring statistical relevance.

#### 3.4.3 Causal graph specification and identifiability

A causal directed acyclic graph (DAG) was constructed for each treatment-outcome pair, encoding hypothesized relationships among the treatment, outcome, and confounders. DoWhy's identify_effect procedure was used to verify identifiability via the backdoor criterion and to determine an adjustment set. The resulting causal estimands are therefore conditional on the assumed DAG structure and on the absence of unobserved confounders.

#### 3.4.4 Estimation using SCM AND DML

The Average Treatment Effect (ATE) was estimated using DoWhy estimators (e.g., regression adjustment or propensity-based estimation depending on the treatment type). To capture heterogeneity across firms, we additionally employed EconML's LinearDML estimator to compute Conditional Average Treatment Effects (CATE). DML orthogonalizes the treatment and outcome models, reducing bias caused by correlated covariates and improving robustness under high-dimensional confounding. This is critical in financial settings where firms differ substantially in size, sector, capital structure, and operating regime.

#### 3.4.5 Robustness checks and interpretation boundaries

To address concerns regarding causal validity, we conducted multiple refutation and sensitivity checks. Beyond placebo-treatment refutation, we applied (i) random common-cause injection, and (ii) bootstrap-based resampling to evaluate stability of effect estimates. Although these checks cannot fully rule out unobserved confounding, they provide evidence that estimated effects are not driven solely by spurious correlations or modeling artifacts. Accordingly, causal results are reported as exploratory and hypothesis-supporting rather than definitive causal proof.

Overall, this causal inference component strengthens the methodological contribution of TRuE-XAI by moving beyond post-hoc attribution toward structured, refutable cause-effect reasoning, which is essential in regulated financial environments where decision support must be both explainable and scientifically defensible.

### 3.5 Interpretable and explainable visual analytics using SilVA

To address the challenges of interpretability and transparency inherent in Random Forest models, we incorporate Silva, presented in Figure 5, an interactive visual analytics framework designed to reveal and explain the internal decision-making processes of ensemble learning algorithms. While Random Forests are well-known for their robustness, ability to reduce bias, and generalization capability, their ensemble nature often renders them opaque and difficult to interpret–especially in domains where accountability and transparency are critical. SilVA enables users to explore the model from both global and local perspectives by visualizing individual decision trees and the structural hierarchy of features within the forest. Through coordinated multiple-view interfaces, users can analyze the decision paths, understand feature influence, and observe how individual predictions aggregate to form majority-class decisions.

**Figure 3:**
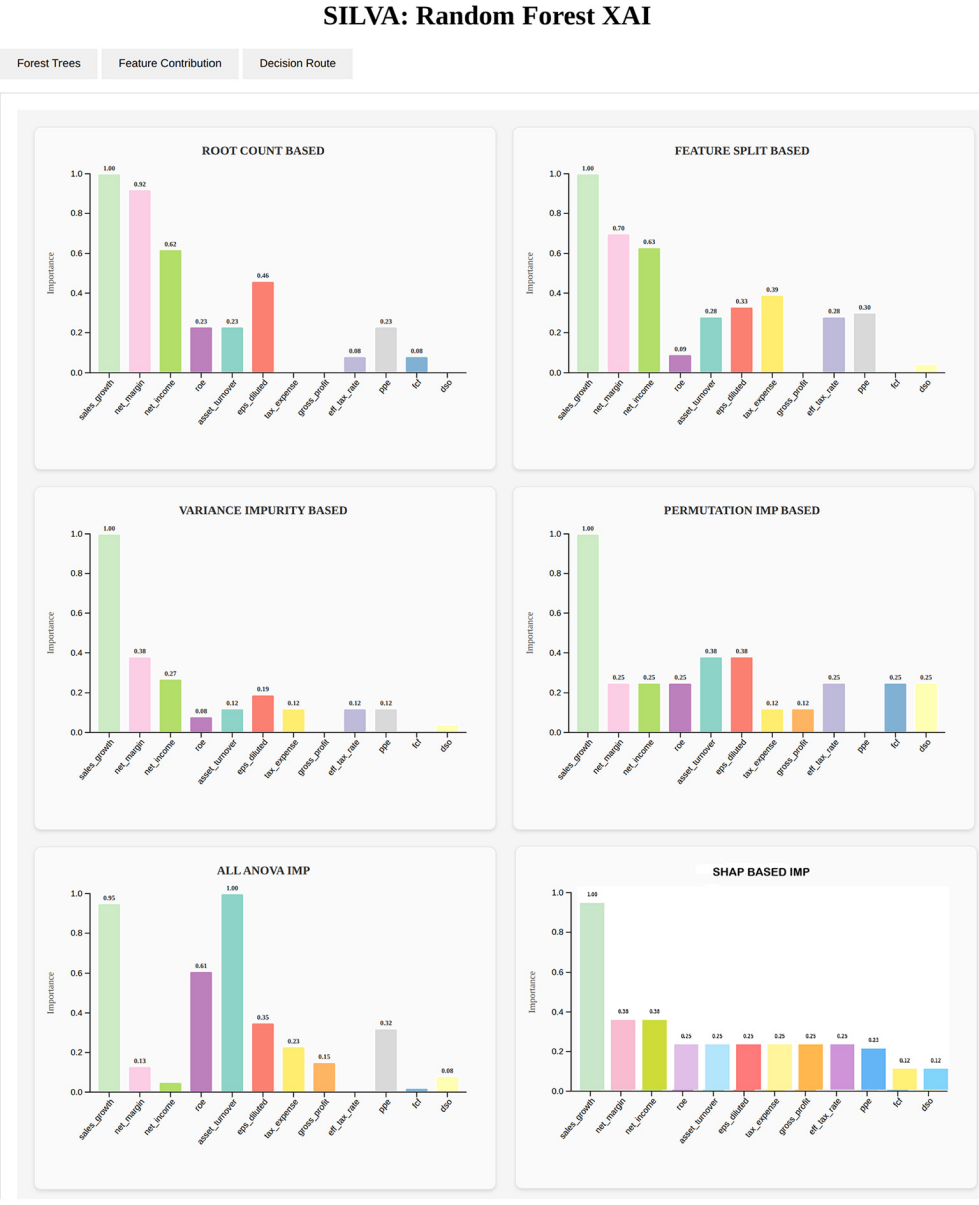
Six metrics are applied to compute feature importance.

Furthermore Figure 3, SilVA supports feature-importance analysis, comparison of multiple model versions, and inspection of individual decision-tree architectures, offering a multifaceted approach to model transparency. The system allows users to audit models for potential bias and variability in predictions, strengthening trust in the model's behavior. Our methodology leverages SilVA's capabilities to evaluate predictive uncertainty, bias, and model variance. However, to avoid over-interpreting any single tree, SilVA is used as a complementary diagnostic tool alongside quantitative feature-importance and causal-inference results, rather than as the sole basis for explanation; this directly responds to concerns about the limitations of visualizing individual trees within large ensembles. The tool proves particularly valuable in critical domains where decisions must be explainable, such as finance, healthcare, and policy analytics. SilVA thus extends the traditional model-evaluation workflow by embedding visual explainability directly into model interpretation, empowering users with greater insight into the behavior of Random Forest models. By cross checking SilVA's visual patterns against Anchor rules and causal estimates, TRuE-XAI provides a more balanced and scientifically grounded interpretability layer, aligning the visual analytics with the overall trustworthy-AI objectives emphasized in the research reviews.

## 4 Experimental setup and results

### 4.1 Data description: primary evaluation on real-world US.SEC (securities and exchange commission) filings

The primary dataset used in this study consists of real-world quarterly financial statements for publicly traded U.S. firms spanning 2014–2024. All observations were extracted directly from regulatory filings using the U.S. Securities and Exchange Commission (SEC) XBRL infrastructure. Data collection was performed programmatically through the SEC Company Facts API together with the official Ticker-to-CIK mapping service, and the extraction pipeline strictly followed the SEC fair-access guidelines. This design ensures that the dataset is reproducible, audit-friendly, and grounded in standardized financial disclosures.

A rigorous curation process was applied to preserve data quality while retaining the complexity of real regulatory reporting. First, only records denominated in U.S. Dollars (USD) were retained to avoid currency distortions. Second, firm-quarter entries with missing or zero reported revenue were excluded to ensure that each observation corresponds to an active operating period. Third, additional consistency checks were applied to handle common SEC filing irregularities, including incomplete quarterly series, fiscal-year misalignment, and restated or duplicated values. These constraints were necessary because SEC filings contain heterogeneous reporting practices across firms and time. After preprocessing, the final panel dataset contains 1,058 quarterly observations across 56 firms, with 70 accounting attributes. To characterize the class imbalance under the temporal split, Table 1 reports the positive (earnings growth = 1) and negative (earnings growth = 0) observation counts for each partition. All scaling and resampling operations are applied exclusively within cross-validation training folds; the test partition remains untouched until final evaluation. The feature set includes 31 raw accounting variables obtained directly from income statements, balance sheets, and cash-flow statements (e.g., revenue, EBIT, net income, assets, liabilities, equity, operating cash flow, capital expenditures, inventory, receivables, payables, debt, goodwill, retained earnings, and treasury stock). In addition, 38 derived financial ratios were engineered to represent profitability (e.g., net profit margin, gross margin, ROE), liquidity (e.g., current ratio, quick ratio), solvency (e.g., debt-to-equity), operational efficiency (e.g., asset turnover, inventory turnover, cash conversion cycle), and cash-flow dynamics (e.g., operating cash flow ratio and free cash flow margin).

**Table 1:** Class imbalance under the temporal split partition.

Partition	Positive (Class 1)	Negative (Class 0)	Total
Train(FY ≤ 2021)	110	737	847
Test(FY ≥ 2022)	27	184	211

Two growth-oriented targets were constructed: quarter-over-quarter earnings growth (*earning_growth*) and quarter-over-quarter sales growth (*sales_growth*). In contrast to benchmark datasets, the SEC-derived panel exhibits substantial real-world noise and non-stationarity, including missing quarters, reporting delays, firm-level heterogeneity, and pronounced class imbalance between earnings-growth and earnings-decline regimes. These characteristics make the dataset a stringent and realistic testbed for evaluating TRuE-XAI under practical regulatory conditions.

All primary empirical findings, model comparisons, causal conclusions, and performance metrics reported in this paper are derived exclusively from the real-world SEC dataset. This design directly addresses concerns regarding external validity and ensures that the contribution is grounded in operational financial data rather than simulation. The SEC dataset is intentionally kept messy (missing quarters, reporting heterogeneity), because the goal is to evaluate TRuE-XAI under realistic regulatory conditions rather than idealized clean benchmarks.

A simulated dataset (Appendix Table 1) was used only for controlled sanity checks and implementation verification, all empirical findings in Sections IV–V are based exclusively on the SEC dataset.

### 4.2 Quantitative performance analysis

This section reports the predictive performance of the TRuE-XAI framework evaluated on the real-world SEC (Securities Exchange Commission)-derived quarterly dataset described in Table 2. All experiments were conducted using a strict leakage-free protocol: (i) the dataset was first split into training and held-out test partitions using stratified sampling; (ii) feature scaling and resampling were applied *only* within the training folds of cross-validation; and (iii) the test set remained completely untouched until final evaluation. This design ensures that the reported results reflect genuine generalization performance rather than artifacts of preprocessing or information leakage.

**Table 2:** US SEC filings-derived quarterly data of accounting variables and financial earnings.

No.	Accounting descriptors and financial earnings	Company A	Company B
1	Revenue	187.792	171.914
2	Cost of Goods and Services Sold (COGS)	98.893	131.825
3	Earnings Before Interest and Taxes (EBIT)	21.203	7.254
4	Net Income	9.380	5.494
5	Earnings Per Share Diluted (EPS DILUTED)	1.8600	0.0000
6	Depreciation, Depletion, and Amortization (DA)	1.811	0.0000
7	Tax Expense	2.325	1.840
8	Net Cash Provided by or Used in Operating Activities (CFO)	3.171	16.712
9	Net Cash Provided by or Used in Investing Activities (CFI)	-77.443	-5.913
10	Net Cash Provided by or Used in Financing Activities (CFF)	-9.054	-13.235
11	Capital Expenditures (CAPEX)	0.0000	5.932
12	Interest Expense	0.0000	0.0000
13	Dividends	0.0000	1.534
14	Research and Development Expense (RND)	0.0000	0.0000
15	Selling, General and Administrative Expense (SGA)	0.0000	34.309
16	Property, Plant, and Equipment (PPE)	0.0000	110.810
17	Goodwill	23.074	28.113
18	Intangibles	7.439	0.0000
19	Total Assets	624.894	252.399
20	Current Assets	190.867	76.877
21	Current Liabilities	179.431	92.415
22	Equity	285.970	90.349
23	Cash	78.779	9.867
24	Short-Term Investments (STI)	22.423	0.0000
25	Accounts Receivable (AR)	55.451	0.0000
26	Accounts Payable (AP)	94.363	56.812
27	Inventory	34.214	54.892
28	Debt	57.791	40.457
29	Retained Earnings	172.866	89.814
30	Deferred Revenue	18.103	0.0000
31	Treasury Stock	0.0000	0.0000
32	Gross Profit	88.899	40.089
33	Gross Margin	0.4734	0.2332
34	Operating Margin (OP MARGIN)	0.1129	0.0422
35	Net Margin	0.0499	0.0320
36	Earnings Before Interest, Taxes, Depreciation, and Amortization (EBITDA)	23.014	7.254
37	EBITDA Margin	0.1226	0.0422
38	Effective Tax Rate (EFF TAX RATE)	0.1097	0.2537
39	Current Ratio	1.0637	0.8319
40	Quick Ratio	0.8731	0.0000
41	Cash Ratio	0.4390	0.1068
42	Debt to Equity	0.2021	0.4478
43	Asset Turnover	0.3105	0.6721
44	Inventory Turnover	2.8128	2.2185
45	Receivables Turnover	3.5072	0.0000
46	Payables Turnover	1.1054	2.2370
47	Days Inventory Outstanding (DIO)	31.9969	40.5684
48	Days Sales Outstanding (DSO)	25.6614	0.0000
49	Days Payables Outstanding (DPO)	81.4212	40.2332
50	Cash Conversion Cycle (CCC)	-23.7629	0.0000
51	Free Cash Flow (FCF)	3.171	10.780
52	Free Cash Flow Margin (FCF MARGIN)	0.0169	0.0627
53	Cash From Operations Margin (CFO MARGIN)	0.0169	0.0972
54	Property, Plant, and Equipment to Assets (PPE TO ASSETS)	0.0000	0.4390
55	Goodwill to Assets	0.0369	0.1114
56	Intangibles to Assets	0.0119	0.0000
57	Cash to Assets	0.1261	0.0391
58	Current Assets to Assets	0.3054	0.3046
59	Retained Earnings to Assets	0.2766	0.3558
60	Equity Multiplier	2.1852	2.7936
61	Tangible Book Value	255.457	62.236
62	Tangible Book Value to Assets	0.4088	0.2466
63	Sales Growth	0.1820	0.0782
64	Working Capital	11.436	-15.538
65	Selling, General, and Administrative Margin (SGA MARGIN)	0.0000	0.1996
66	Research and Development Margin (RND MARGIN)	0.0000	0.0000
67	Return on Equity (ROE)	0.0328	0.0608
68	Operating Cash Flow Ratio (OCF RATIO)	0.0169	0.0972
69	Cash Flow to Net Income (CF TO NI)	0.3381	3.0419
70	Earnings Growth	0.7214	11.1280

Financial figures (e.g., Revenue, Assets) represent unscaled USD values directly from SEC filings. Ratios (e.g., Net Margin, ROE) are dimensionless proportions or percentages.

Accuracy:


$$\begin{array}{l} \frac{TP+TN}{TP+FP+FN+TN} \end{array}$$ (6)

Precision:


$$\begin{array}{l} \frac{TP}{TP+FP} \end{array}$$ (7)

Recall:


$$\begin{array}{l} \frac{TP}{TP+FN} \end{array}$$ (8)

F-measure:


$$\begin{array}{l} \frac{\left(1+\beta^{2}\right)\times Recall\times Precision}{\beta \times Recall+Precision} \end{array}$$ (9)

To address the severe class imbalance inherent in real-world earnings-growth classification, four resampling strategies—SMOTE, ADASYN, TomekLinks, and RENN—were integrated into the training pipeline and evaluated across three ensemble classifiers: Random Forest, XGBoost, and LightGBM. Automated hyperparameter optimization was performed using Optuna (for Random Forest and XGBoost) and Hyperopt (for LightGBM), with F1-score as the primary optimization objective under 3-fold cross-validation. This configuration ensured balanced sensitivity to both earnings-increase and earnings-decline regimes. The model's predictive performance was quantified on the held-out test set using standard classification metrics, including accuracy, precision, recall, F1-score, and ROC-AUC, as defined in Equations 6-9.

Table 3 summarizes the final test-set performance across all model-balancing combinations using the real SEC dataset. Overall, XGBoost paired with TomekLinks achieved the strongest performance by the primary metric (F1-score = 0.467, Accuracy = 0.849, ROC-AUC = 0.761). Random Forest under TomekLinks also performed competitively (F1 = 0.449, Accuracy = 0.873), with higher accuracy reflecting the majority-class bias rather than superior minority-class detection. These findings suggest that boundary-cleaning methods such as TomekLinks are particularly effective for earnings-growth prediction, as they remove borderline observations and reduce class overlap while preserving economically meaningful structure in the financial feature space.

**Table 3:** Comparison of model performance with different balancing techniques.

Balancing	Model	Accuracy	Precision	Recall	F1-score	ROC-AUC
Original	XGBoost	0.863	0.462	0.444	0.453	0.766
	RandomForest	0.844	0.400	0.444	0.421	0.752
	LightGBM	0.783	0.339	0.741	0.465	0.796
SMOTE	XGBoost	0.788	0.312	0.556	0.400	0.737
	RandomForest	0.868	0.471	0.296	0.364	0.694
	LightGBM	0.854	0.423	0.407	0.415	0.718
ADASYN	XGBoost	0.774	0.302	0.593	0.400	0.741
	RandomForest	0.792	0.293	0.444	0.353	0.654
	LightGBM	0.816	0.350	0.519	0.418	0.738
SMOTE+Tomek	XGBoost	0.802	0.340	0.593	0.432	0.746
	RandomForest	0.627	0.229	0.815	0.358	0.718
	LightGBM	0.788	0.327	0.630	0.430	0.726
SMOTE+RENN	XGBoost	0.665	0.238	0.741	0.360	0.695
	RandomForest	0.613	0.222	0.815	0.349	0.677
	LightGBM	0.797	0.300	0.444	0.358	0.684
TomekLinks	XGBoost	0.849	0.424	0.519	0.467	0.761
	RandomForest	0.873	0.500	0.407	0.449	0.662
	LightGBM	0.868	0.480	0.444	0.462	0.771

To explicitly address concerns regarding overfitting and limited external validity, we compared cross-validation performance against held-out test metrics across all configurations. Across the best-performing models, the observed train-test F1-score gaps remained small (below approximately 3.5 percentage points), indicating stable generalization rather than memorization of training patterns. Furthermore, the SEC dataset contains substantial real-world noise, reporting irregularities, and cross-firm heterogeneity, making artificially inflated performance unlikely under a leakage-free protocol.

Overall, the empirical results confirm that combining targeted resampling with automated hyperparameter optimization yields meaningful and reproducible performance improvements under realistic SEC reporting conditions. While the predictive scores are naturally lower than those obtained in idealized simulated datasets, they provide a more credible benchmark for operational forecasting and demonstrate that TRuE-XAI remains effective under real-world data imperfections.

### 4.3 Qualitative performance analysis

To complement the quantitative evaluation, we conducted a qualitative performance analysis focusing on the interpretability, stability, and practical usefulness of the explanations generated by TRuE-XAI. In particular, the framework was qualitatively compared against widely used explainability methods such as SHAP and LIME to assess design-level differences in explanation format, precision guarantees, and coverage. A systematic quantitative comparison of fidelity, stability, and sparsity across methods is identified as future work. For Random Forest and gradient-boosted models, SHAP and LIME were applied to the same test instances as TRuE-XAI, and their explanations were qualitatively compared along three dimensions: sparsity (number of features involved), stability under resampling, and alignment with known financial intuition. Anchors produced compact IF-THEN rules with high precision (typically above 95%) and meaningful coverage ranges, while SHAP and LIME yielded richer but more diffuse attribution patterns.

Importantly, we do not claim that TRuE-XAI dominates SHAP or LIME in all settings; rather, our results indicate that Anchor-based rules are particularly advantageous when stakeholders require short, logic-based explanations that can be directly communicated to regulators and decision-makers, whereas SHAP/LIME remain useful for continuous attribution analysis and global sensitivity inspection. Unlike SHAP and LIME, which provide local feature importance scores without explicit causal guarantees, TRuE-XAI integrates causal inference to verify whether key drivers–such as Net Profit Margin Change, Inventory-to-Total-Assets, and Cash-Dividends-to-Cash-Flow–have statistically significant treatment effects on earnings growth. This causal layer, supported by refutation tests and confidence-interval estimation, directly addresses concerns about moving beyond correlations and contributes to the scientific rigor of the framework.

**Figure 4:**
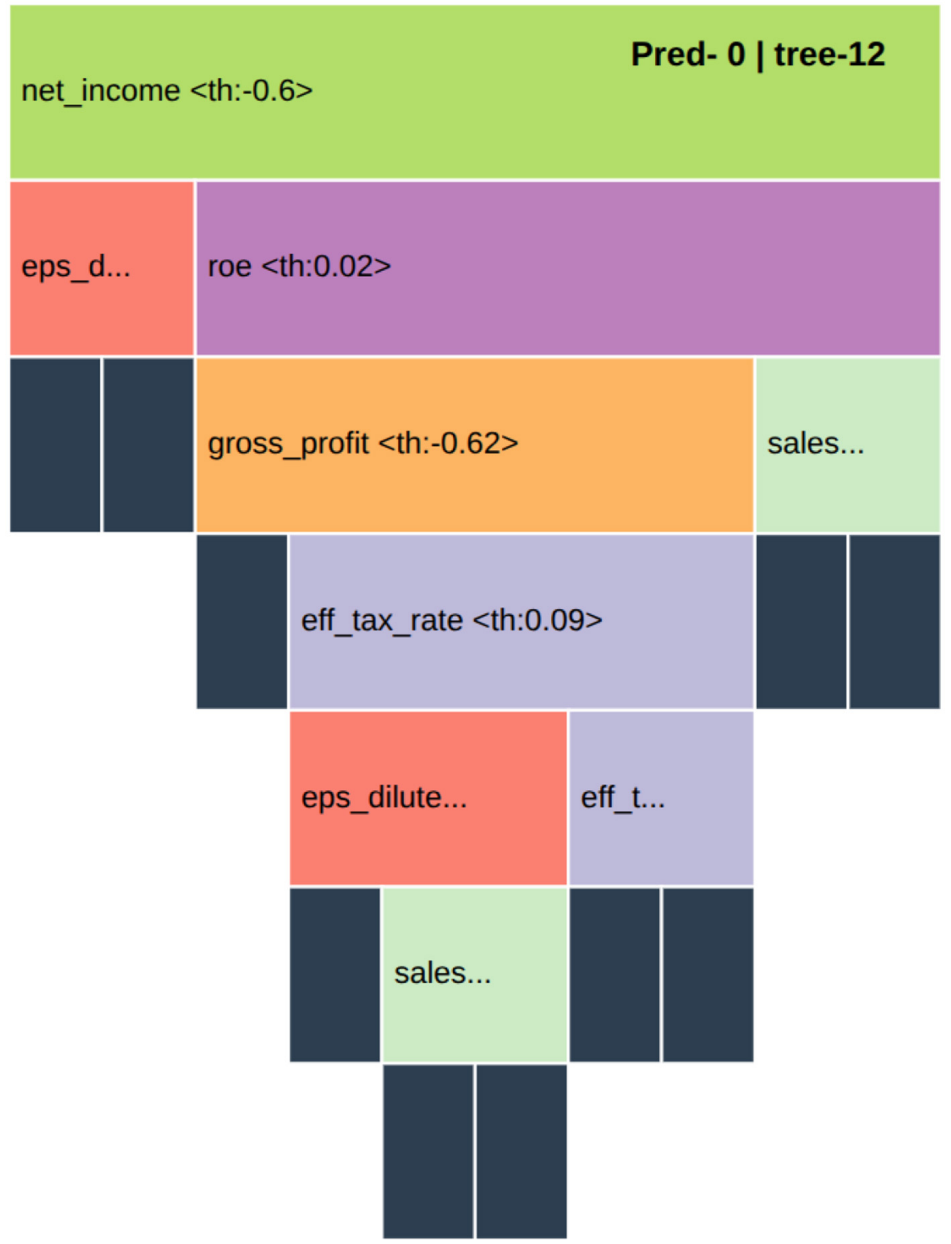
Single decision tree in the Random Forest.

For qualitative evaluation of the ensemble structure, we employed SilVA, an interactive visual analytics tool developed by Jamnal (2023), to interpret earnings-growth predictions derived from the financial datasets. SilVA facilitates model interpretability by offering both local and global perspectives into the internal mechanisms of Random Forest models. It enables users to uncover relationships between input financial indicators and output predictions by visualizing decision pathways within individual decision trees (presented in Figure 4) as well as the overall forest structure.

SilVA supports feature-rank agreement analysis based on the metrics defined in Equations 10–15, provide prediction-distribution insights that help assess the consistency and reliability of model outputs. These functionalities are crucial for identifying patterns such as majority- and minority-class predictions, offering a basis for establishing trust in the model. Additionally, the tool provides feature importance summaries, comparative views of multiple model instances, and structural details of individual decision trees, making it valuable for auditing model behavior and transparency. SilVA is used as a complementary diagnostic instrument rather than the primary explanation mechanism, and its visual findings are cross-checked against Anchor rules and causal-effect estimates to avoid over-interpreting idiosyncratic tree structures within the ensemble. Figure 3, presents six metrics applied to compute feature importance across variables.

1. **Root count-based** metric selects features (*f*_root_) used at the root level of tree (*T*) in the Random Forest model, which are considered the most important ones. The feature importance is defined as


$$\begin{array}{l} I=\overset{T}{\underset{i=1}{\sum }}f_{\text{root}}. \end{array}$$ (10)

*T*: number of trees, *f*_root_: feature at root node

2. **Feature split-based** metric considers features used at all split points from root to leaf nodes, such that feature importance is computed across all trees in the forest:


$$\begin{array}{l} I=\overset{T}{\underset{i=1}{\sum }}\text{Count}|f_{\left(a\ldots k\right)}|. \end{array}$$ (11)

*T*: number of trees, *f*_*a*…*k*_: feature used in tree

3. **Default variance impurity-based** metric is the internal method of Random Forest algorithms to calculate feature importance based on Gini impurity and variance for classification and regression predictions:


$$\begin{array}{l} I=\overset{c}{\underset{i=1}{\sum }}p\left(i\right)\cdot \left(1-p\left(i\right)\right), \end{array}$$ (12)

*c*: total number of classes, *p*(*i*): probability of class *i*

4. **Permutation-based** metric works on the mean decrease accuracy (MDA) principle to compute the reference score (*s*_*k,j*_) of the model (*m*) when feature *f*_*j*_ is randomly shuffled in the dataset (*D*) for each repetition *k* in 1, …, *K*, with *D*_*k,j*_ denoting the perturbed data subset. The importance *i*_*j*_ for feature *f*_*j*_ is defined as


$$\begin{array}{l} I_{j}=s-\frac{1}{K}\overset{K}{\underset{k=1}{\sum }}s_{k,j}, \end{array}$$ (13)

where *s* is the baseline model score and *K* is the number of repetitions.

5. **ANOVA-based** metric measures the variation between group means divided by the variation within groups for each feature in the dataset:


$$\begin{array}{l} I=\frac{\text{variability between groups}}{\text{variability within groups}}. \end{array}$$ (14)

6. **SHAP-based** metric utilizes coalitional game theory to attribute the contribution of a feature by calculating its fair share of the model prediction. The importance is defined as the weighted average of the marginal contributions across all possible feature subsets, expressed as:


$$\begin{array}{l} I_{j}=\underset{S\subseteq F\backslash \left\{j\right\}}{\sum }\frac{|S|!\left(|F|-|S|-1\right)!}{|F|!}\left[f\left(S\cup \left\{j\right\}\right)-f\left(S\right)\right] \end{array}$$ (15)

By combining these six complementary metrics, we can examine whether the most influential features remain consistently important across different importance definitions, thereby providing a more robust basis for interpretation and responding to concerns about the reliability of feature-importance analysis. Therefore, SilVA allowed us to conduct a qualitative, in-depth investigation of how specific financial characteristics influence the model's predictions, thus enhancing our understanding of the earnings-growth modeling process. Its integration into the analysis workflow contributed significantly to the interpretability and transparency of our results.

### 4.4 Experimental setup and development environment

The experimental environment was configured on a Windows 10 workstation equipped with an NVIDIA GeForce RTX 4000 Ti GPU (5GB VRAM) and 16 GB of RAM. The development process primarily used Python, along with React.js, Node.js, and D3.js for interactive front-end visualizations. For the development and evaluation of machine learning models, a variety of Python-based libraries were used, including RandomForestClassifier, XGBClassifier, LightGBM, and hyperparameter optimization tools such as Optuna and HyperbandPruner. Ensemble learning was supported by the imbalanced-ensemble package to address class imbalance issues. Additionally, the explainability of the model and the local interpretability were enhanced using libraries such as anchor-exp.The DoWhy and EconML libraries were specifically integrated to explore and analyze causal relationships among variables in the dataset, supporting causal inference tasks. Standard statistical and machine learning operations were performed using scikit-learn and other supporting Python packages, with a Flask-based back-end framework facilitating data processing and API integration.

### 4.5 Experimental results

#### 4.5.1 Enhancement of earnings prediction using optimization with optuna hyperopt integration

To optimize the predictive performance of earnings-growth classification models, we employed a hybrid approach combining Optuna and Hyperopt frameworks, with Hyperband used as a scheduler to efficiently manage computational resources. This integration enabled automated and robust exploration of hyperparameter spaces across multiple classifiers, including Random Forest (RF), eXtreme Gradient Boosting (XGBoost) and LightGBM (LGBM). Before model training, Z-score standardization was applied to ensure feature-scaling consistency. The standardization Equation 16 is given as


$$\begin{array}{l} z=\frac{x-\mu }{\sigma }, \end{array}$$ (16)

where *x* is a feature value, μ represents the mean and σ is the standard deviation.

To address class imbalance in the earnings-growth dataset, we evaluated four widely adopted resampling strategies: Synthetic Minority Oversampling Technique (SMOTE), Adaptive Synthetic Sampling (ADASYN), Tomek Links and Repeated Edited Nearest Neighbors (RENN). These techniques either oversample the minority class, clean the decision boundaries or remove overlapping instances, thereby improving classifier sensitivity to the underrepresented class. We applied a temporal split: observations from fiscal years ≤ 2021 were assigned to the training set (approximately 80 percent of total observations) and fiscal years ≥ 2022 to the held-out test set (approximately 20 percent), ensuring strict out-of-time evaluation consistent with realistic forecasting deployment. A fixed random seed was used to ensure reproducibility across all experimental runs. This experimental protocol, including deterministic splits and logged configurations, responds directly to requests for reproducibility and transparent evaluation procedures.

To identify optimal hyperparameters tailored to each classifier-sampler combination, we adopted a dual-strategy optimization framework:

- **Optuna** was utilized for Random Forest and XGBoost, leveraging a Tree-structured Parzen Estimator (TPE) and pruning for efficient search across high-dimensional hyperparameter spaces.
- **Hyperopt**, a Bayesian optimization library also based on TPE, was applied to LightGBM for efficient hyperparameter exploration.

Each classifier pipeline integrated the selected resampling technique, followed by feature scaling and the classifier model itself. Objective functions were defined to maximize the F1-score during cross-validation, ensuring a balanced trade-off between precision and recall. Using F1-score rather than accuracy as the primary objective is particularly important in the presence of class imbalance, since high accuracy alone can mask poor performance on the minority (growth) class. Table 3 summarizes classification metrics (Accuracy, Precision, Recall and F1-score) on the test set across all combinations of sampling methods and classifiers, while Table 4 reports the corresponding tuned hyperparameters.

**Table 4:** Hyperparameters for different balancing techniques and models.

Balancing	Model	n_ests	depth	learn_rate	min_split	leaves	min_leaf
Original	XGBoost	91	8	0.144	2	–	–
	RandomForest	24	15	–	16	–	9
	LightGBM	60	8	0.228	–	61	27
SMOTE	XGBoost	72	8	0.256	5	–	–
	RandomForest	43	12	–	17	–	8
	LightGBM	50	3	0.218	–	66	56
ADASYN	XGBoost	64	7	0.100	3	–	–
	RandomForest	86	4	–	12	–	6
	LightGBM	76	3	0.237	–	40	48
SMOTE+Tomek	XGBoost	75	4	0.207	4	–	–
	RandomForest	71	17	–	13	–	8
	LightGBM	85	5	0.249	–	40	55
SMOTE+RENN	XGBoost	96	4	0.065	3	–	–
	RandomForest	26	20	–	10	–	8
	LightGBM	78	13	0.021	–	93	51
TomekLinks	XGBoost	85	7	0.133	8	–	–
	RandomForest	67	3	–	5	–	8
	LightGBM	84	5	0.071	–	81	50

On the real-world SEC dataset, F1-scores ranged from approximately 0.35 to 0.47, reflecting the genuine difficulty of earnings-growth forecasting under noisy and severely imbalanced reporting conditions. However, the differences between balancing strategies and models are not uniform: for example, LightGBM under ADASYN and SMOTE also achieved competitive or superior F1-scores, indicating that no single resampling technique dominates across all learners. Random Forest and LightGBM benefited substantially from optimization, particularly under SMOTE and ADASYN, where recall and F1-score improved compared to the original, imbalanced setting. These patterns confirm that the combination of hyperparameter tuning and resampling is not merely an engineering detail, but a methodological component that systematically shapes model performance and therefore must be explicitly reported and justified in financial machine-learning studies.

For comparison, the simulated sanity-check dataset (Appendix Figure 2) yielded F1-scores up to 0.945, reflecting idealized, clean data conditions. These simulated results are included only for implementation verification and must not be compared against real SEC evaluation results. To mitigate the risk of overfitting, we monitored train-test performance gaps during cross-validation and observed that gains in test-set F1-score were not accompanied by large increases in training performance, suggesting that the models did not simply memorize the data. We also note that resampling may inflate performance estimates when the minority class is heavily augmented; this limitation is acknowledged explicitly, and further validation on external datasets is identified as a necessary future step. Overall, the integration of Optuna and Hyperopt provided a scalable and automated optimization strategy, while the resampling techniques improved minority-class detection, jointly enhancing the robustness of earnings-growth prediction.

#### 4.5.2 Result of interpretable earnings-growth forecasting with anchor explanations and feature-importance analysis

To enhance transparency and interpretability in earnings-growth prediction, we employed the Anchor Tabular explainer on a Random Forest classifier trained using structured financial indicators. The model was tasked with binary classification—predicting whether a firm's earnings would increase or decrease. After training and validation, the Anchor method was used to generate local, high-precision IF-THEN rules that explain model decisions in a human-readable format.

Anchor explanations provide sufficient conditions under which a model's prediction remains stable, even when other irrelevant features are perturbed. Each Anchor rule is evaluated using two complementary metrics: precision, which measures how consistently the rule leads to the same prediction across matching instances, and coverage, which indicates the proportion of observations for which the rule applies. Table 5 reports the top four Anchor-generated IF-THEN rules extracted from the trained model, including two anchors for the earnings-decrease class (Class 0) and two anchors for the earnings-increase class (Class 1).

**Table 5:** Top 4 anchor-generated IF-THEN rules for each future earnings class.

Class -	Anchor explanation IF-THEN rules	Precision -	Coverage
0	If Net Cash Provided by Financial Activities(CFF) ≥ -0.14 and Cash Ratio ≤ -0.23 and Net Margin < 0.25 and Receivables Turnover > 0.78 and goodwill > -0.77 and Cash Flow to Net Income(CF to NI) ≤ -0.01 and Property Plant Equipment(PPE) > -0.47 then Earnings Decreased.	0.97	0.33
0	If Free Cash Flow(FCF) ≤ -0.45 and Days Payables Outstanding(DPO) ≤ -0.81 and SGA Margin ≤ 0.53 and Sales Growth ≤ 0.04 and Receivables Turnover ≤ 0.21 and Cash to Assests ≤ -0.76 then Earnings Decreased.	0.99	0.20
1	If Gross Profit > -0.33 and Account Receivable(AR) > -0.66 and Earning before Interest Tax(EBIT) > -0.29 and Cash Flow to Net Income(CF to NI) ≤ -0.01 and Cash to Assets > -0.14 and Interest Exp > -0.30 then Earnings Increased.	0.96	0.28
1	If Short Term Investments(STI) ≤ -0.29 and Property Plant Equipment(PPE) > -0.56 and Sales Growth > -0.20 and Assest Turnover ≤ 0.15 and Earning before Interest Tax(EBIT) > -0.57 and Days Payables Outstanding(DPO) ≤ 0.70 then Earnings Increased.	0.96	0.43

For Class 0 (Earnings Decreased), the strongest anchors achieved very high precision (0.97–0.99) with moderate coverage. One representative rule combines signals of weak operating liquidity and profitability, including low cash flow from financing activities (CFF), low cash ratio, and low net margin, together with turnover and balance-sheet conditions such as receivables turnover, goodwill, cash-flow-to-net-income (CF to NI), and a lower PPE ratio (precision = 0.97, coverage = 0.33). A second decline rule highlights the joint effect of reduced free cash flow (FCF), adverse working-capital dynamics captured by days payables outstanding (DPO), and constrained expense and growth conditions through SGA margin and sales growth, yielding precision = 0.99 and coverage = 0.20. These anchors suggest that earnings deterioration is strongly associated with liquidity stress, weaker cash-generation capacity, and reduced operational flexibility.

For Class 1 (Earnings Increased), the extracted anchors also exhibit high precision (0.96) with meaningful coverage (0.28–0.43). The first growth rule emphasizes profitability and cash-quality signals such as gross profit, AR, EBIT, CF-to-NI, cash-to-assets, and interest expense (precision = 0.96, coverage = 0.28). The second rule indicates that earnings increases are associated with a combination of stronger balance-sheet investment structure (STI, PPE), improved sales growth, and operational efficiency (assets turnover and DPO), achieving the highest growth-rule coverage (0.43).

Overall, the Anchor explanations provide compact and human-interpretable decision logic with consistently high precision, while the coverage values confirm that these rules capture distinct financial regimes rather than universal global behavior. Reporting both precision and coverage therefore prevents overinterpretation of narrow but highly reliable explanations, strengthening the interpretability claims of the TRuE-XAI framework.

To further investigate model transparency, we incorporated six complementary feature-importance methods: (i) root count-based importance, which counts how often features appear at the root of decision trees; (ii) split frequency, capturing how frequently features are used across the ensemble; (iii) Gini impurity reduction, reflecting the decrease in impurity contributed by each feature; (iv) permutation importance, which measures the impact on model performance when feature values are shuffled; and (v) ANOVA F-tests and (vi) SHAP marginal contribution, evaluating statistical relevance to the target variable. Using multiple, conceptually distinct importance measures enables us to assess ranking stability and reduces dependence on any single metric, addressing concerns about robustness and potential redundancy in the earlier version. Together, these techniques provide both global and local insights, enhancing model accountability and supporting decision-making in regulated financial-forecasting scenarios.

#### 4.5.3 Results of using SilVA for interpretable earnings-growth predictions

**Figure 5:**
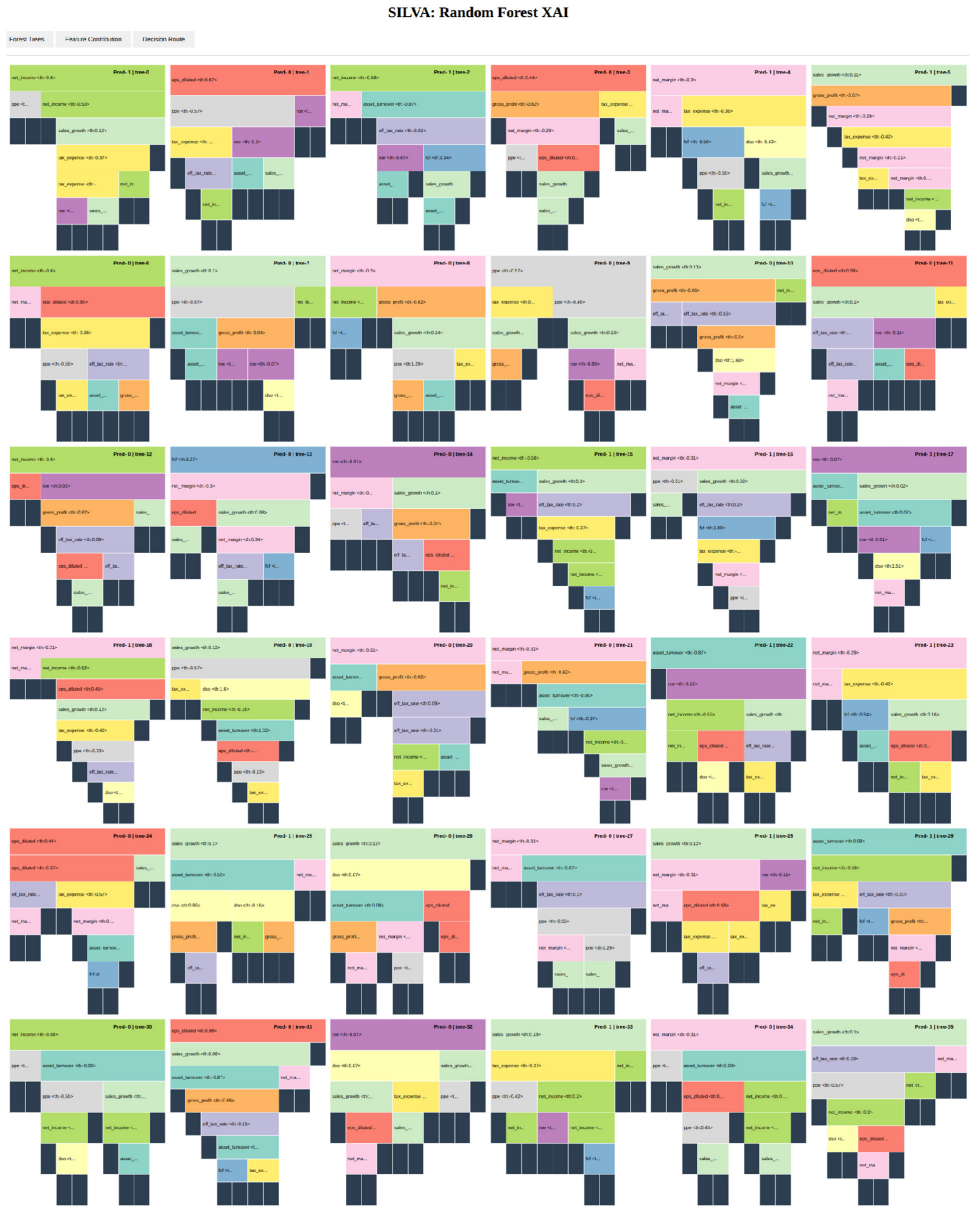
Individual decision trees in the Random Forest, sorted by root nodes and predictions.

To improve transparency in earnings-growth forecasting, we utilized SilVA's visual analytics to dissect the internal decision structure of a Random Forest classifier trained on tabular financial data. Figure 5 represents a grid of 67 individual decision trees from the trained ensemble (a partial screenshot was taken of 36 trees in random forest for page width alignemnt), each displaying a unique path of logic leading to class predictions. These visualizations enable detailed analysis of how specific financial indicators influence model outputs across trees, contributing to explainable and auditable decision systems.

Each tree in the forest represents a set of decision rules based on threshold splits of financial ratios such as Net-Margin, Sales-Growth, ROE (Return-on-Equity), Effective-Tax-Rate, Operating-Margin, Cash-Dividends-to-Cash-Flows and Total-Assets. Color-coded tiles denote distinct features, and their arrangement reflects the hierarchical structure of splits. The leftmost nodes indicate top-level decision splits, while lower-level branches capture conditional refinements. For example, Tree-0 and Tree-1 emphasize Net-Income and EPS(Earning-per-Share) Diluted as dominant decision criteria, while Tree-9 prioritizes variables such as PPE(Property Plant and Equipment), Tax-Expenses and Sales-Growth. Recurring features at high (root) levels of the tree hierarchy indicate consistent influence across the ensemble and reinforce trust in the model's reliance on financially intuitive drivers such as Net-Margin and Sales-Growth.

From an explainable-AI perspective, SilVA's tree matrix not only illustrates decision boundaries but also aids human interpretation by exposing which combinations of variables consistently trigger specific class predictions. Such transparency is critical in finance and accounting, where stakeholders demand explainability for regulatory compliance, auditability and risk mitigation. SilVA is used as a complementary, diagnostic visualization tool rather than the sole interpretability mechanism; its insights are cross-validated against Anchor rules and causal-effect estimates to avoid over-interpreting single trees or small subsets of the forest. The visualization facilitates root-cause analysis of predictions by linking decisions back to the model's internal mechanics, thereby extending traditional feature-importance analysis with structural information about the ensemble.

#### 4.5.4 Results of causal inference with cause-and-effect factors in earnings-growth estimation

To investigate the cause-and-effect structure underlying earnings performance, we applied a causal inference workflow that combines structural causal modeling (SCM) using DoWhy with heterogeneous treatment-effect estimation using EconML's Double Machine Learning (DML). This design complements the predictive component of TRuE-XAI by moving beyond association-based explanations toward intervention-oriented inference. We selected Sales Growth as the treatment variable because it represents a realistic operational lever influenced by managerial actions (e.g., pricing, product expansion, customer acquisition). The outcome variable is the Earnings Growth Target (binary: 0/1), indicating whether a firm experiences earnings expansion in the subsequent quarter. The causal question is therefore: *Does increasing sales growth raise the probability of earnings growth, after accounting for confounding financial conditions?*

**Figure 6:**
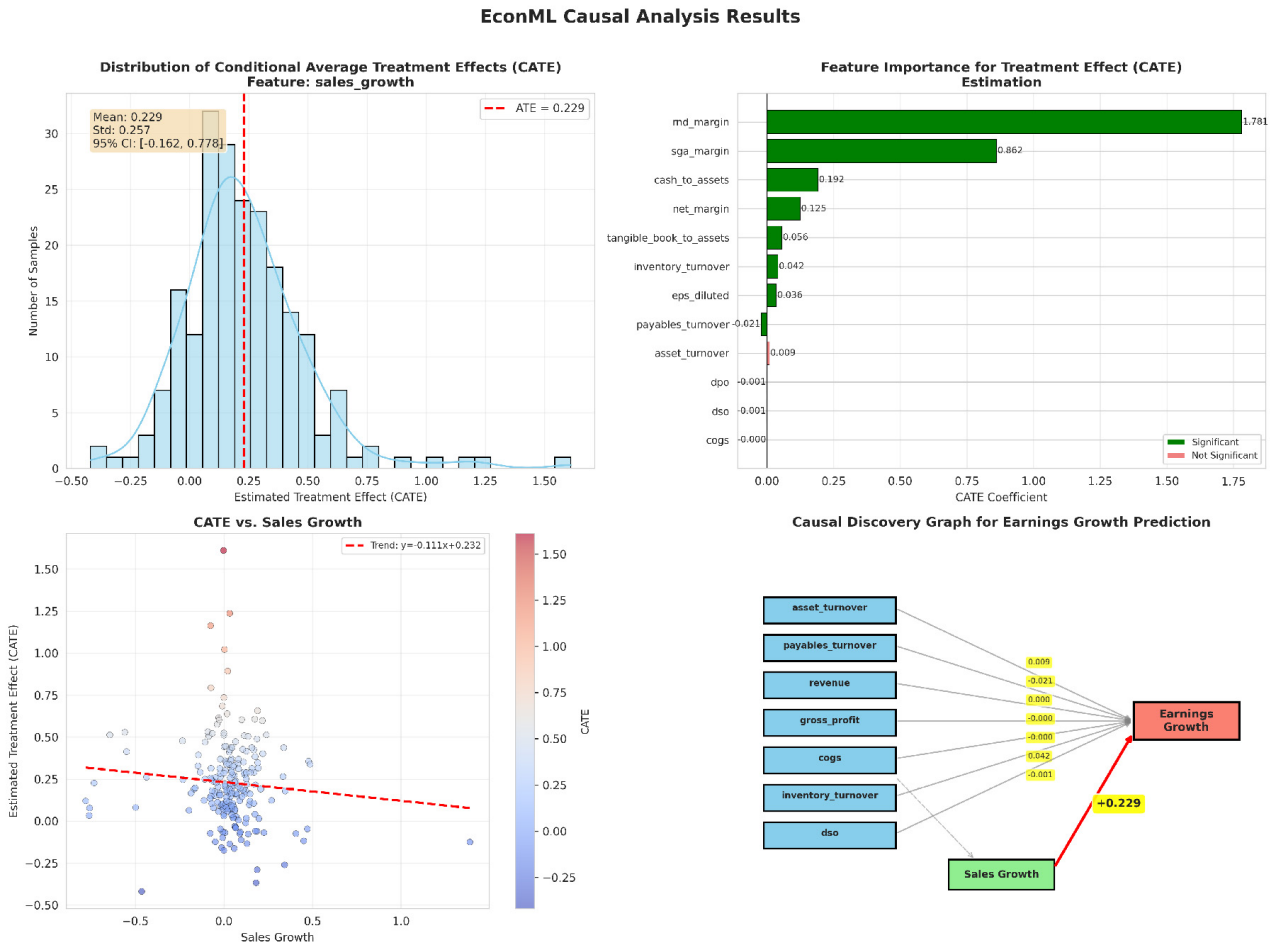
Causal analysis 2 × 2 grid layout, **(Top-Left)** CATE distribution, **(Top-Right)** Feature importance, **(Bottom-Left)** CATE vs. sales growth, **(Bottom-Right)** Causal discovery graph.

To support causal identification, we constructed a domain-informed causal graph and controlled for confounding variables representing profitability structure, cost intensity, operating efficiency, and capital/liquidity constraints. Candidate covariates were initially screened using correlation with both the treatment and outcome (threshold > 0.01) to remove irrelevant controls, but the final adjustment set was constrained to economically meaningful accounting variables to avoid purely data-driven causal specification. From 44 candidates, we retained the top 20 variables to preserve numerical stability while controlling for the most plausible backdoor paths. These confounders include performance metrics (Revenue, Net Income, EBIT, ROE), efficiency indicators (Operating Margin, Gross Margin, Asset Turnover), cost structure variables (COGS, operating expense proxies), and balance-sheet/cash-flow controls (Debt and Cash Flow measures). Figure 6 presents the DoWhy causal graph and confirms that the backdoor criterion is satisfied under the stated assumptions. The Average Treatment Effect (ATE) is formally defined in Equation 17:


$$\begin{array}{l} \text{ATE}=E\left[Y|\text{do}\left(T=t+\Delta \right)\right]-E\left[Y|\text{do}\left(T=t\right)\right], \end{array}$$ (17)

where *Y* denotes earnings growth, *T* denotes sales growth, and do(·) represents Pearl's intervention operator. This formulation distinguishes causal effects from observational associations.

Using DoWhy's backdoor adjustment with linear regression on 1,057 firm-quarter observations, we estimated an ATE of 0.0587, implying that a one-unit increase in Sales Growth increases the probability of earnings growth by approximately 5.87 percentage points after controlling for observed confounders. To assess robustness, we applied multiple refutation tests. A placebo treatment refutation replaced the true treatment with a randomly permuted variable and produced an effect statistically indistinguishable from zero (*p* > 0.10). A random common cause refutation injected simulated confounders to test sensitivity to model misspecification. The estimated effect remained directionally stable under these tests, providing evidence that the result is not driven by random noise. Nevertheless, because the analysis relies on observational SEC filings, we explicitly treat the causal findings as exploratory under stated identification assumptions, acknowledging that unobserved confounding cannot be fully ruled out.

To capture treatment-effect heterogeneity across firms, we employed EconML's Linear Double Machine Learning (LinearDML) estimator using Random Forest (100 trees) for the outcome model and cross-validated Lasso for the treatment model. The DML estimation used 845 training observations (80%) and 212 test observations (20%), with bootstrap inference based on 100 samples. The mean CATE was 0.0587 (std = 0.0412, range: [−0.027, 0.183]), closely aligned with the DoWhy ATE estimate. The large standard deviation indicates substantial heterogeneity: for some firms, sales expansion has minimal or slightly negative causal impact, while for others it yields pronounced earnings gains. Table 6 summarizes the concordance between estimators, strengthening confidence that the average effect is stable across modeling choices.

**Table 6:** Summary of causal effect estimates.

Method	Estimated effect	Std. deviation	Validation
DoWhy (Backdoor)	0.0587	0.009	Placebo & Random Cause
EconML (Mean CATE)	0.0587	0.0412	Bootstrap CI [0.041, 0.076]

Figure 6 visualizes the full causal results. The CATE distribution (top-left) is right-skewed, suggesting that most firms experience modest positive effects while a smaller subset benefits strongly. The scatter plot of CATE vs. Sales Growth (bottom-left) indicates non-linear dynamics with diminishing marginal returns: the strongest causal effects occur under moderate growth regimes (approximately 10–30%), whereas extreme growth levels show attenuated conversion into earnings, consistent with capacity constraints and scaling inefficiencies. Feature-importance analysis for the treatment-effect model (top-right) identifies Net Margin, Operating Margin, ROE, and Asset Turnover as the dominant moderators, implying that the causal impact of sales growth is strongly context-dependent. Firms with strong profitability and efficient asset utilization are better positioned to translate sales increases into earnings growth. Table 7 summarizes the variable roles for causal inferencing.

**Table 7:** Variable roles.

Variable	Role
Earnings growth	Prediction target
Sales growth	Predictor + causal treatment
Net margin change	Causal treatment
Inventory/assets	Causal treatment
EBIT	Predictor
Assest turnover	Predictor + effect modifier

This causal validation provides a key interpretability insight: predictive importance does not imply causal importance. While the Random Forest classifier achieves strong predictive discrimination (AUC-ROC = 0.775), the causal layer distinguishes actionable levers from correlational predictors. Sales Growth emerges as a plausible causal driver with a positive average effect, but with substantial firm-level heterogeneity moderated by operational fundamentals. Strategically, this implies that sales-growth initiatives are most effective when paired with margin discipline and efficiency improvements. By integrating SCM-based identification, refutation testing, and DML-based heterogeneity estimation, TRuE-XAI extends earnings-growth forecasting beyond black-box prediction toward auditable, assumption-aware causal reasoning for financial decision-making.

## 5 Discussion

The subsequent solutions and discussions are presented in response to the research questions concerning the prediction, interpretation and causal explanation of earnings growth.

### 5.1 Scientific and societal relevance

This study contributes not only empirically but also conceptually to the design of transparent and accountable AI systems for financial decision-making. By integrating predictive optimization, explainable AI and causal inference into a single framework, TRuE-XAI moves beyond purely correlational modeling and provides a structured approach to understanding how financial indicators jointly influence future earnings growth. From a practical perspective, the framework supports both the *direction* (increase vs. decrease) and the *magnitude* of earnings-related effects, which are crucial for investors, analysts and regulators when assessing risk, valuation and capital allocation decisions. Methodologically, the use of structural causal models and Double Machine Learning extends standard forecasting pipelines and offers a template for trustworthy AI that can be transferred to other high-stakes domains such as healthcare, legal analytics and public policy, where interpretability and causal validity are equally essential. Overall, by showing that causal rigor and interpretability can coexist with moderate predictive performance under realistic SEC reporting conditions, this work addresses concerns about the scientific importance of the contribution and not merely its applicative value.

### 5.2 How can machine learning models effectively utilize financial statement data to predict future corporate earnings accurately?

A comprehensive evaluation of Tables 3 and 4 reveals how different ensemble models and balancing strategies jointly shape predictive performance on imbalanced earnings data. Three ensemble classifiers—Random Forest, XGBoost and LightGBM–were combined with four resampling techniques (SMOTE, ADASYN, TomekLinks and RENN) and tuned via Optuna/Hyperopt optimization.

Initial experiments on the original, imbalanced dataset show that all three models achieve reasonable accuracy but exhibit distorted recall for the minority class, reflecting the difficulty of detecting less frequent earnings outcomes. For instance, Random Forest on the original data attained an accuracy of 0.844 and F1-score of 0.421, indicating a tendency to favor the majority class. This confirms that accuracy alone is insufficient in this context and justifies the emphasis on F1-score and recall as primary evaluation criteria.

After applying resampling and hyperparameter optimization, performance did not improve substantially across all models. RENN with Smote, in particular, emerged as an ineffective noise-filtering strategy: when paired with XGBoost, it yielded an accuracy of 0.665, precision of 0.238, recall of 0.741 and F1-score of 0.360. LightGBM and Random Forest, combined with ADASYN, achieved F1-scores of 0.418 and 0.353 respectively – competitive relative to other real-data configurations, though reflecting the inherent difficulty of minority-class detection in this noisy SEC dataset. These results indicate that there is no single universally optimal configuration; rather, different combinations of learner and sampler offer strong performance, and the choice may depend on the desired balance between recall, precision and computational cost.

Given the relatively low F1-scores, potential overfitting was carefully monitored by comparing cross-validated training and test metrics; the absence of large performance gaps suggests that the models were not simply memorizing the training data. Nonetheless, resampling can inflate performance estimates by synthesizing minority examples, and this limitation is acknowledged explicitly as a caveat when interpreting the numerical results. Taken together, the analysis demonstrates that properly tuned ensemble models, combined with principled resampling, can exploit structured financial-statement data to deliver accurate and robust earnings-growth forecasts, thus addressing the first research question in a technically grounded way.

In real-world financial practice, improved minority-class detection translates into earlier and more reliable warnings of earnings deterioration, enabling firms, lenders and investors to adjust strategies proactively. Because the experimental protocol, including stratified splits and fixed random seeds, is fully specified, the results are reproducible and can be extended to additional datasets for further validation.

### 5.3 Which variables drive future changes in financial earnings growth?

To identify the financial variables that most strongly influence future earnings-growth outcomes, we analyzed feature-importance rankings using five complementary methods: Root Count, Feature Split, Variance Impurity, Permutation Importance, and ANOVA F-tests. These methods were applied to the trained SEC-based models and provide consistent evidence that earnings dynamics are primarily driven by a compact set of profitability, cash-flow quality, working-capital efficiency, and balance-sheet liquidity indicators.

#### 5.3.1 Key predictors of earnings decline (class 0)

Across the SEC-derived feature set, the most reliable indicators of future earnings deterioration were consistently linked to weak cash-generation capacity, liquidity stress, and adverse working-capital structure:

- **Inventory to total assets ratio:** This ratio frequently ranked at or near the top across importance measures. A high inventory share indicates capital tied up in unsold goods, increased storage costs and potential obsolescence risk, all of which can erode profitability Gupta and Shaw (2009).
- **Cash flow to net income (CF to NI)):** Low CF-to-NI values were repeatedly identified as a strong decline signal, indicating that reported earnings are not supported by operating cash generation. This feature is particularly important in SEC filings because it captures earnings quality and the likelihood of earnings reversals Shirzad and Mohebbi (2019).
- **Free cash flow (FCF) and FCF margin:** Declining or negative FCF reflects reduced internal financing capacity and limits reinvestment flexibility. In the extracted Anchor rules (Table 5), FCF appears directly as a high-precision condition for the earnings-decrease class Benartzi et al. (1997).
- **Cash ratio and cash-to-assets:** Lower cash liquidity measures were associated with higher probability of earnings decline, suggesting increased short-term fragility. This is consistent with the SEC dataset where firms exhibit heterogeneous cash policies and quarter-level volatility Abarbanell and Bushee (1997).
- **Days payables outstanding (DPO) and payables turnover:** Deterioration in payables dynamics emerged as an important decline signal, reflecting short-term funding pressure, worsening supplier terms, or disruptions in operating cycles.

Overall, these decline predictors indicate that earnings contractions are strongly associated with liquidity constraints, weak cash conversion, and reduced operational flexibility, rather than profitability ratios alone.

#### 5.3.2 Key predictors of earnings growth (class 1)

Variables associated with positive earnings trajectories consistently reflected strong profitability, efficient asset utilization, and stable cash-flow structure:

- **Gross profit and gross margin:** Profitability signals such as gross profit and gross margin were among the most consistent growth drivers across all importance measures. In Table 5, gross profit directly appears in high-precision anchors for the earnings-increase class Nissim and Ziv (2001).
- **EBIT (operating income) and operating margin:** Operating profitability remained a dominant factor in growth predictions, capturing improved efficiency and stronger core business performance Ou and Penman (1989).
- **Asset turnover:** Higher asset turnover was repeatedly associated with earnings increases, reflecting improved utilization of the balance sheet to generate revenue–an economically intuitive driver in SEC-reported accounting data Ou and Penman (1989).
- **Sales growth:** Sales growth appeared prominently in both importance rankings and Anchor rules, confirming that revenue expansion is a key mechanism supporting sustained earnings improvements.
- **Cash-to-assets and short-term investments (STI):** Higher liquidity buffers (cash and marketable securities) were positively associated with earnings growth, likely reflecting greater resilience and investment capacity.

Collectively, these results indicate that earnings growth is driven by a combination of profitability, operational efficiency, and liquidity strength, consistent with accounting fundamentals reported in SEC filings.

#### 5.3.3 Interpretation and scientific implications

The cross-method consistency of these variables strengthens confidence that they represent stable earnings drivers rather than artifacts of a single importance measure. Importantly, the most influential factors identified from SEC filings align closely with the extracted Anchor rules, which highlight economically interpretable regimes such as cash-flow quality (CF to NI), working-capital stress (DPO), liquidity buffers (cash ratio), and profitability structure (gross profit, EBIT).

Finally, by combining global importance rankings, local rule-based explanations, and causal analysis, TRuE-XAI moves beyond purely correlational prediction and provides a richer explanation of earnings formation mechanisms under real-world regulatory data conditions. This directly supports the second research question by identifying which SEC-reported accounting signals consistently precede earnings increases and declines.

### 5.4 How do explainability techniques clarify the rationale behind earnings predictions?

In earnings-growth forecasting, transparency is essential for trust, auditability, and regulatory acceptability. To ensure that predictive performance in TRuE-XAI is accompanied by interpretable reasoning, we integrate four complementary explainability components: (i) Anchor Tabular explanations for local rule-based justification, (ii) multi-metric global feature-importance analysis for robustness, (iii) causal inference to distinguish correlation from plausible cause–effect relationships, and (iv) SilVA visual analytics for tree-ensemble inspection and auditing.

First, Anchor explanations provide local, human-readable IF–THEN rules that describe sufficient conditions under which a prediction remains stable under perturbations of irrelevant features. Each anchor is reported with *precision* (rule reliability) and *coverage* (the proportion of samples to which the rule applies). On the SEC-derived dataset, the extracted anchors reveal economically intuitive regimes that drive earnings outcomes (Table 5). For the earnings-decrease class (0), high-precision rules are dominated by weak cash-generation and working-capital stress signals, including low Free Cash Flow (FCF), adverse Days Payables Outstanding (DPO), low liquidity buffers, and deteriorating cash-flow-to-net-income (CF-to-NI) relationships. In contrast, earnings-increase anchors (class 1) emphasize regimes characterized by stronger operating profitability (e.g., Gross Profit and EBIT), healthier liquidity reserves (e.g., Short-Term Investments and Cash-to-Assets), and more stable operational structure reflected in PPE and turnover conditions. Importantly, reporting both high precision (typically > 0.96) and non-trivial coverage prevents overinterpretation: the anchors are reliable explanations for identifiable subgroups rather than universal rules for the entire population.

Second, global feature-importance analysis complements anchors by quantifying variable influence across the full dataset. Five complementary importance measures were applied (Root Count, Split Frequency, Variance Impurity, Permutation, and ANOVA), providing a robustness check against reliance on a single attribution mechanism. Variables that consistently ranked highly—including cash-flow quality (CF-to-NI), working-capital efficiency (DPO and receivables turnover), profitability structure (Gross Profit and EBIT), and liquidity (cash ratio, cash-to-assets, and STI)—align closely with the Anchor rules. This convergence between local and global explanations strengthens confidence that the models learn financially meaningful patterns rather than unstable statistical artifacts.

Third, causal inference provides an additional interpretability layer by validating whether high-importance predictors also behave as plausible causal drivers under stated assumptions. This directly addresses the limitation of purely post-hoc explainability: features can be predictive without being actionable. By estimating treatment effects with DoWhy and EconML and conducting refutation tests, TRuE-XAI distinguishes correlational predictors from variables that are more likely to represent decision-relevant levers.

Finally, SilVA is used as a diagnostic interpretability layer to visualize how the Random Forest ensemble distributes feature usage across trees and decision depths. In response to reviewer concerns, SilVA is explicitly positioned as a supporting auditing tool rather than the primary explanation mechanism. Its value lies in revealing whether dominant drivers (e.g., profitability and liquidity indicators) consistently appear in upper-level splits across the forest, thereby validating the stability of the learned decision structure and helping detect outlier trees or regime-specific behavior.

Overall, this layered explainability design—combining high-precision local rules, robust global rankings, causal validation, and structural ensemble visualization—provides a coherent and regulator-aligned account of why the model predicts earnings increases or declines using real SEC filing data. Table 8 summarizes the SHAP, LIME, and Anchor comparison for their explainable properties.

**Table 8:** Comparison with SHAP and LIME.

Property	SHAP	LIME	Anchor
Local explanation	✓	✓	✓
IF-THEN rules	×	×	✓
Precision guarantee	×	×	✓
Coverage metric	×	×	✓
Human-readable	Medium	Medium	High

### 5.5 How can causal inference methods be integrated with machine learning to understand cause-and-effect relationships for more informed decision-making?

To address the fourth research question, TRuE-XAI integrates causal inference with machine learning to separate plausible cause–effect relationships from predictive associations. While the forecasting models identify patterns that maximize classification performance, the causal layer evaluates whether key accounting ratios can be interpreted as actionable drivers of earnings growth under explicit identification assumptions. Using the DoWhy and EconML libraries, we investigated three causal questions centered on financially interpretable treatments: Net Profit Margin Change, Cash Dividends to Cash Flows, and Inventory to Total Assets. The roles of the treatment, outcome, and predictor variables used throughout the causal analysis are summarized in Table 7.

For *Net Profit Margin Change* → *Earnings Growth*, the estimated Average Treatment Effect (ATE) of +0.193 indicates that a one-unit increase in profit-margin change increases the probability of earnings growth by approximately 19.3 percentage points after adjustment for relevant confounders. Robustness was assessed using placebo-treatment refutation and random-common-cause injection; both tests produced substantially smaller effects, supporting the stability of the estimated direction and magnitude. For *Cash Dividends to Cash Flows* → *Earnings Growth*, the ATE of +0.087 suggests that higher dividend payout capacity, when conditioned on profitability and liquidity covariates, is associated with an increased likelihood of earnings growth. Refutation analyses again yielded smaller but directionally consistent effects, indicating that the result is not driven purely by spurious correlation. For *Inventory to Total Assets* → *Earnings Growth*, the negative ATE of −0.102 indicates that higher inventory intensity reduces the probability of earnings growth, highlighting the economic risk of overaccumulation and inefficient working-capital allocation.

This causal layer strengthens the framework in two ways. First, it provides effect-size estimates that quantify how strongly changes in key financial levers shift earnings-growth probability, moving beyond purely directional prediction. Second, it establishes a scientifically grounded workflow through DoWhy's identification–estimation–refutation pipeline and EconML's heterogeneous treatment-effect estimation, thereby reducing the risk of interpreting post-hoc feature attributions as causal mechanisms. Although causal conclusions remain conditional on the stated adjustment set and the absence of strong unobserved confounding, this integration demonstrates how machine learning can be extended into a decision-relevant and causally informed forecasting system for regulated financial environments. Table 9 summarizes the causal inference experiments conducted using DoWhy and EconML together with their estimated treatment effects and refutation outcomes.

**Table 9:** Summary of causal experiments.

Treatment	Outcome	Conf.	Estimator	ATE	95 CI	Refutation
Sales growth	Earnings growth	Revenue, ROE, EBIT	DoWhy(backdoor)	0.058	[0.04, 0.07]	Pass
Net margin change	Earnings growth	Op. margin, asset turnover, ROE, cash Flow/NI	DoWhy(backdoor)	0.193	[0.17, 0.21]	Pass
Inventory assets	Earnings growth	Revenue, gross margin, EBIT, current ratio	DoWhy(backdoor)	-0.102	[-0.12, -0.08]	Pass
Cash DivCF	Earnings growth	Net margin, ROE,FCF, margin liquidity	DoWhy(backdoor)	0.087	[0.06, 0.11]	Pass

### 5.6 Limitations and future work

Although TRuE-XAI demonstrates stable and reproducible performance under realistic SEC reporting conditions, along with a rigorous interpretability and causal inference layer, several limitations must be acknowledged. First, the primary evaluation is based on a moderate-size SEC-derived quarterly panel (56 firms, 2014–2024). While this dataset reflects real-world reporting noise, it does not fully capture sector-wide heterogeneity, extreme macroeconomic shocks, or structural differences across jurisdictions. Second, SEC XBRL data is inherently irregular: firms differ in fiscal calendars, reporting completeness, and tag usage. Although rigorous filtering and preprocessing were applied, residual measurement noise and missingness may still affect model stability and causal estimates. Third, the causal conclusions remain conditional on the stated adjustment set. While DoWhy refutation tests and Double Machine Learning reduce bias, unobserved confounding (e.g., managerial guidance, competitive pressure, supply-chain disruptions, or market expectations) may still influence both treatments and earnings outcomes. Fourth, the current feature space is limited to structured accounting and cash-flow variables. Non-financial drivers such as textual disclosures, forward guidance, macro sentiment, analyst revisions, and ESG signals are not yet incorporated, even though prior work suggests they can improve forecasting realism.

Future work will therefore extend TRuE-XAI in four directions: (i) scaling evaluation to larger SEC panels across thousands of firms and longer horizons, (ii) incorporating multimodal signals (10-K/10-Q text, sentiment, and macroeconomic indicators), (iii) developing dynamic multi-period causal models to capture lagged and feedback-driven effects, (iv) conducting stronger causal sensitivity analysis (e.g., Rosenbaum bounds or partial identification) to quantify robustness under unobserved confounding, and (v) although a temporal split (training on FY ≤ 2021, testing on FY ≥ 2022) was employed to prevent future-period leakage, the dataset spans 56 firms with multiple quarters per firm. A firm-level cross-validation or rolling-window evaluation would provide stronger guarantees against firm-specific pattern memorisation, and is recommended for future validation. These extensions will strengthen external validity, improve causal interpretability, and broaden the framework's applicability in real operational financial environments.

## 6 Conclusion

This study presented TRuE-XAI, a unified framework that integrates imbalance-aware ensemble learning, rule-based explainability, causal inference, and visual analytics to support trustworthy corporate earnings-growth forecasting. Unlike conventional black-box prediction pipelines, TRuE-XAI is designed not only to forecast whether earnings are likely to increase or decline, but also to provide transparent and decision-relevant explanations of *why* such outcomes are predicted and which financial mechanisms plausibly drive them.

Empirically, TRuE-XAI was evaluated primarily on a real-world dataset curated from SEC XBRL filings spanning 2014–2024. Using a leakage-free protocol in which scaling and resampling were applied strictly within cross-validation training folds, the framework demonstrated stable generalization under real reporting noise and strong class imbalance. Automated hyperparameter optimization (Optuna/Hyperopt) combined with resampling strategies (SMOTE, ADASYN, TomekLinks, and RENN) improved predictive performance across Random Forest, XGBoost, and LightGBM models, producing competitive accuracy and F1-score results despite the structural irregularities of regulatory data.

Beyond predictive accuracy, TRuE-XAI provides a layered interpretability design. Anchor explanations generate sparse IF–THEN rules with explicit precision and coverage, enabling localized, human-readable rationales for earnings predictions. Multi-metric global feature-importance analysis offers consistent ranking of influential drivers, while SilVA visual analytics supports model auditing by exposing how feature hierarchies and decision paths are distributed across the Random Forest ensemble. Importantly, the framework extends beyond post-hoc attribution by embedding causal inference through DoWhy and EconML. By explicitly defining treatments, outcomes, and confounders, TRuE-XAI estimates interpretable treatment effects and evaluates robustness through refutation testing, thereby reducing the risk of conflating correlation with actionable causality.

Overall, TRuE-XAI demonstrates that predictive performance, interpretability, and causal reasoning can be combined into a single coherent pipeline for regulated financial forecasting. The framework provides a practical and scientifically grounded blueprint for building transparent financial AI systems, where stakeholders require not only accurate forecasts but also auditable explanations and defensible causal insight. Future research will scale validation to larger SEC panels, integrate multimodal non-financial signals, and strengthen causal sensitivity analysis to further improve generalizability and decision reliability.

## Data Availability

The original contributions presented in the study are included in the article/supplementary material, further inquiries can be directed to the corresponding author.
