# Supplementary material for: TRuE-XAI: causal and explainable ai framework for trustworthy corporate earnings growth forecasting
Source: Front Artif Intell. 2026 Jul 27;9:1885910. doi: 10.3389/frai.2026.1885910 (PMC13454063; doi:10.3389/frai.2026.1885910)
Supplement: Supplementary file 1 [file Supplementary_file_1.pdf]

1 APPENDIX

Simulated Dataset for Implementation Verification purpose Only

To verify implementation correctness under controlled conditions, we additionally generated a small simulated dataset following the empirical accounting structure of Ou and Penman. This dataset is included solely as an internal sanity-check resource (e.g., validating derived-ratio computations, debugging the preprocessing pipeline, and confirming causal-estimation code behavior under simplified assumptions). It is not used for any primary evaluation, model selection, performance reporting, or substantive empirical conclusions. All predictive results, model comparisons, and causal findings reported in the main manuscript are based exclusively on the real-world SEC-derived dataset.

Table S1: This appendix provides implementation-level validation material included for reproducibility and code verification only, and it does not contribute to the primary empirical findings of the paper.

No.	Accounting descriptors and financial earnings	Company A	Company B
1	Inventory_to_Total_Assets	0.4967	-1.1063
2	Net_Profit_Margin_Change	-0.1383	-1.1962
3	Cash_Dividends_to_Cash_Flows	0.6477	0.8125
4	Dividend_per_Share_Change	1.5230	1.3562
5	Operating_Profit_to_Sales_Change	-0.2342	-0.0720
6	Gross_Margin_Ratio	-0.2341	1.0035
7	Equity_to_Fixed_Assets	1.5792	0.3616
8	Pretax_Income_to_Sales	0.7674	-0.6451
9	Times_Interest_Earned	-0.4695	0.3614
10	Sales_to_Total_Assets	0.5426	1.5380
11	Asset_Turnover	-0.4634	-0.0358
12	Debt_to_Equity_Ratio	-0.4657	1.5646
13	Current_Ratio	0.2420	-2.6197
14	Quick_Ratio	-1.9133	0.8219
15	Return_on_Assets	-1.7249	0.0870
16	Return_on_Equity	-0.5623	-0.2990
17	Total_Assets_Growth	-1.0128	0.0918
18	Total_Liabilities_to_Assets	0.3142	-1.9876
19	Operating_Cash_Flow	-0.9080	-0.2197
20	Free_Cash_Flow	-1.4123	0.3571
21	Cash_Ratio	1.4656	1.4779
22	Interest_Coverage_Ratio	-0.2258	-0.5183
23	Net_Income_to_Sales	0.0675	-0.8085
24	Working_Capital_Ratio	-1.4247	-0.5018
25	Earnings_Before_Tax	-0.5444	0.9154
26	Earnings_Before_Interest_Tax	0.1109	0.3288
27	Market_to_Book_Ratio	-1.1510	-0.5298
28	Price_to_Earnings_Ratio	0.3757	0.5133
29	Price_to_Sales_Ratio	-0.6006	0.0971
30	Price_to_Cash_Flow	-0.2917	0.9686
31	Net_Income_Growth	-0.6017	-0.7021

Continued on next page...

Table S1 – Continued from previous page

No.	Accounting descriptors and financial earnings	Company A	Company B
32	Revenue_Growth	1.8523	-0.3277
33	Gross_Profit	-0.0135	-0.3921
34	EBITDA_Margin	-1.0577	-1.4635
35	SGA_to_Sales	0.8225	0.2961
36	Effective_Tax_Rate	-1.2208	0.2611
37	Operating_Margin	0.2089	0.0051
38	Capital_Expenditure_to_Sales	-1.9597	-0.2346
39	R&D_to_Sales	-1.3282	-1.4154
40	Inventory_Turnover	0.1969	-0.4206
41	Accounts_Receivable_Turnover	0.7385	-0.3427
42	Accounts_Payable_Turnover	0.1714	-0.8023
43	Days_Inventory_Outstanding	-0.1156	-0.1613
44	Days_Sales_Outstanding	-0.3011	0.4041
45	Days_Payable_Outstanding	-1.4785	1.8862
46	Cash_Conversion_Cycle	-0.7198	0.1746
47	Interest_Bearing_Debt	-0.4606	0.2576
48	Book_Value_per_Share	1.0571	-0.0744
49	Total_Debt_to_EBITDA	0.3436	-1.9188
50	Net_Debt_to_Equity	-1.7630	-0.0265
51	Dividend_Payout_Ratio	0.3241	0.0602
52	Retention_Ratio	-0.3851	2.4632
53	Operating_Income_to_Assets	-0.6769	-0.1924
54	Pretax_Profit_Margin	0.6117	0.3015
55	Net_Margin	1.0310	-0.0347
56	Revenue_per_Share	0.9313	-1.1687
57	Net_Operating_Assets	-0.8392	1.1428
58	Net_Operating_Cash_Flow	-0.3092	0.7519
59	Dividend_Coverage_Ratio	0.3313	0.7910
60	Return_on_Capital_Employed	0.9755	-0.9094
61	Tangible_Book_Value	-0.4792	1.4028
62	Cash_Flow_Coverage_Ratio	-0.1857	-1.4019
63	Earnings_Growth	0.0000	1.0000

Note: All performance, causal, and explainability results reported in this paper are derived from the real-world SEC dataset described in Section IV-A.

Balancing	Model	n_estimators	max_depth	learning_rate	min_samples_split	num_leaves
Original	XGBoost	100	5	0.162	10	
	RandomForest	91	16		2	
	LightGBM	100	4	0.193	30	40
SMOTE	XGBoost	48	4	0.190	7	
	RandomForest	86	19		10	
	LightGBM	150	5	0.178	2	35
ADASYN	XGBoost	75	8	0.156	6	
	RandomForest	71	15		3	
	LightGBM	150	9	0.038	7	37
TomekLinks	XGBoost	65	6	0.162	5	
	RandomForest	68	20		9	
	LightGBM	80	2	0.135	8	38
RENN	XGBoost	59	7	0.0766	4	
	RandomForest	100	5		3	
	LightGBM	100	0	0.086	2	31

Figure S1: Simulated data – Hyperparameters for different balancing techniques and models.

**Balancing**	**Model**	**Accuracy**	**Precision**	**Recall**	**F1-score**
RENN	XGBoost	0.926	0.933	0.950	0.942
	RandomForest	0.922	0.929	0.924	0.921
	LightGBM	0.911	0.901	0.910	0.915
SMOTE	XGBoost	0.911	0.907	0.924	0.914
	RandomForest	0.919	0.925	0.931	0.928
	LightGBM	0.938	0.933	0.931	0.935
ADASYN	XGBoost	0.922	0.924	0.935	0.929
	RandomForest	0.915	0.921	0.931	0.926
	LightGBM	0.945	0.937	0.953	0.945
TomekLinks	XGBoost	0.910	0.908	0.932	0.919
	RandomForest	0.916	0.922	0.928	0.924
	LightGBM	0.934	0.928	0.937	0.933
Original	XGBoost	0.841	0.778	0.950	0.855
	RandomForest	0.748	0.664	0.996	0.795
	LightGBM	0.884	0.812	0.995	0.895

Figure S2: Simulated data – Hyperparameters for different balancing techniques and models.
